# Near‐Infrared Emissive Super Penetrating Conjugated Polymer Dots for Intratumoral Imaging in 3D Tumor Spheroid Models

**DOI:** 10.1002/advs.202403398

**Published:** 2024-07-18

**Authors:** Soner Karabacak, Başak Çoban, Ahu Arslan Yıldız, Ümit Hakan Yıldız

**Affiliations:** ^1^ Department of Chemistry Izmir Institute of Technology Urla Izmir 35430 Turkey; ^2^ Department of Bioengineering Izmir Institute of Technology Urla Izmir 35430 Turkey

**Keywords:** conjugated polymers dots, intratumoral imaging, near‐infrared emission, penetration profile, tumor spheroid models

## Abstract

This study describes the formation of single‐chain polymer dots (Pdots) via ultrasonic emulsification of nonionic donor‐acceptor‐donor type (D–A–D) alkoxy thiophene–benzobisthiadiazole‐based conjugated polymers (Poly BT) with amphiphilic cetyltrimethylammonium bromide (CTAB). The methodology yields Pdots with a high cationic surface charge (+56.5 mV ± 9.5) and average hydrodynamic radius of 12 nm. Optical characterization reveals that these Pdots emit near‐infrared (NIR) light at a maximum wavelength of 860 nm owing to their conjugated polymer backbone consisting of D–A–D monomers. Both colloidal and optical properties of these Pdots make them promising fluorescence emissive probes for bioimaging applications. The significant advantage of positively charged Pdots is demonstrated in diffusion‐limited mediums such as tissues, utilizing human epithelial breast adenocarcinoma, ATCC HTB‐22 (MCF‐7), human bone marrow neuroblastoma, ATCC CRL‐2266 (SH‐SY5Y), and rat adrenal gland pheochromocytoma, CRL‐1721 (PC‐12) tumor spheroid models. Fluorescence microscopy analysis of tumor spheroids from MCF‐7, SH‐SY5Y, and PC‐12 cell lines reveals the intensity profile of Pdots, confirming extensive penetration into the central regions of the models. Moreover, a comparison with mitochondria staining dye reveals an overlap between the regions stained by Pdots and the dye in all three tumor spheroid models. These results suggest that single‐chain D–A–D type Pdots, cationized via CTAB, exhibit long‐range mean free path of penetration (≈1 µm) in dense mediums and tumors.

## Graphical abstract

The single chain near infrared (NIR) emissive Pdots with high cationic surface charge enable penetration in dense medium such as tumor spheroids. Both colloidal and optical properties of Pdots make them promising fluorescent probe in bioimaging.

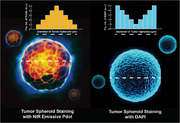

## 1 Introduction

**Scheme 1:**
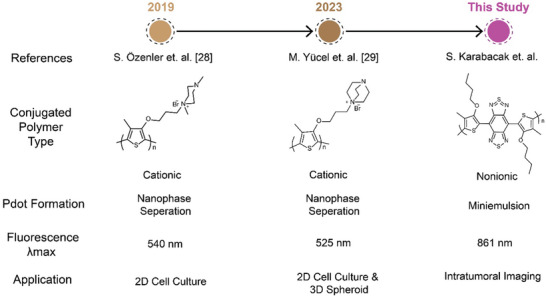
Timeline for this study.

Micro‐alterations or deformation in tissues and their vicinity are often critical, and they are potentially an indication of onsets or progress of diseases.^[^ ^1^, ^2^ ^]^ The current clinical setups relying on microscopy‐associated techniques provide convincing and interpretable images though they have drawbacks due to the resolution of imaging methodology.^[^ ^3^, ^4^ ^]^ The limitations in resolution, as well as artifacts in imaging, may therefore be misleading because they increase the level of uncertainty that conceals such micro‐alterations in denser tissue. The major limitations in fluorescence microscopy imaging appear as lesser penetration of the visible range of electromagnetic radiation (400–700 nm) and autofluorescence of fluorescent probes. In particular, autofluorescence causes an emission in a wider perimeter than the original location of probes and mistargets the region of interest. The necessity for optimization in fluorescence microscopy imaging modalities is evident.^[^ ^5^, ^6^, ^7^ ^]^ NIR fluorescent imaging has recently drawn attention because it offers minimal interference absorption, low biological autofluorescence, and high tissue penetration for biological applications over visible fluorescence imaging.^[^ ^8^, ^9^, ^10^, ^11^ ^]^ To date, semiconducting polymers, rare earth‐doped nanoparticles, quantum dots (CuInS/ZnS), single‐walled carbon nanotubes, carbon dots, and metal nanoclusters have been reported as NIR emissive probes.^[^ ^12^, ^13^, ^14^, ^15^ ^]^ These and other studies demonstrate that the NIR emissive probes have promising potential in bioimaging, diagnosis, photodynamic therapy, and controlled drug delivery due to their very low autofluorescence.^[^ ^16^, ^17^, ^18^, ^19^ ^]^ Among the molecular NIR probes, the semiconducting polymers may undergo controlled condensation/precipitation and yield nanoparticles, so‐called “Pdots,” exhibiting superior emissive and colloidal properties. The fluorescence properties of Pdots based on NIR emissive conjugated polymers allow advanced bioimaging applications through improved photophysical properties such as high brightness, high extinction coefficients, and good photostability.^[^ ^20^, ^21^, ^22^, ^23^, ^24^ ^]^ The size of Pdots appears as a critical parameter that limits or boosts up bio‐imaging modalities; for instance, size of 50 nm Pdots are prone to accumulate in the cytoplasm as well as lysosomes whereas sub−20 nm size Pdots can penetrate to the crowded cellular compartments and mitochondria, as well as to microtubules around the nucleus.^[^ ^21^, ^22^ ^]^ Thus, these ultrasmall Pdots provide better tissue and subcellular penetration in biological studies.^[^ ^25^ ^]^ However, as stated recently, the synthesis of Pdot with a size of ≈10 nm remains a challenge due to colloidal instability as size decreases.^[^ ^26^ ^]^ Even though the conventional methodologies, nanoprecipitation,^[^ ^27^ ^]^ and miniemulsion^[^ ^23^ ^]^ have been reported for preparation of NIR Pdots, the comprehensive study concerning size versus penetration capabilities of Pdots is still missing due to probable colloidal instability problems. Recently, our group has suggested a nanophase separation method for Pdot preparation which facilitates the formation of ultra‐small‐sized Pdots from cationic semiconducting polymers.^[^ ^28^ ^]^ **Scheme** **1** illustrates conjugated polymer based Pdot, their preparation methodologies, optic properties, and area of application. The described nanophase separation method appears to provide monodisperse Pdots with less than 10 nm in size. Özenler et al. have shown that Pdots made of cationic Pdots with 4 nm size exhibited augmented penetration to the nucleus of cancerous liver cells providing sorting of healthy and cancer cells. Later Yücel et al. applied Pdots of cationic polythiophenes to the breast cancer cell line of MCF‐7 and human breast adenocarcinoma cancer cells (estrogen‐independent) (MDA‐MB‐231) and their tumoroid models.^[^ ^29^ ^]^ Differential fluorescence signals were collected from the MDA‐MB‐231 tumoroid models which characterized the translocation of Pdots in the triple‐negative cancer cells. However, nanophase separation is not applicable for neutral(nonionic) semiconducting polymers,^[^ ^30^, ^31^ ^]^ and penetration characteristics of Pdots have not been studied comprehensively in dense medium such as in tumoroids.^[^ ^32^, ^33^ ^]^

Here, we suggest a preparation methodology and investigation of penetration behavior 12 nm sized cationic NIR Pdots consisting of nonionic D–A–D type alkoxy thiophene and benzobisthiadiazole‐based conjugated polymer, which is named as poly(4,8‐di‐3‐bromo‐4 methylthiophenebenzobisthiadiazole) (poly BT) via sonication‐assisted emulsification. The energy provided via ultrasound in the presence of cationic surfactant (CTAB), Poly BT, is a low band gap D–A–D type water insoluble conjugated polymer that is (mini)emulsified to produce positively charged Pdots. Here, CTAB is in the class of lipophilic cation‐generating cationic small‐sized nanoparticles with a surface potential > +40 mV as the other mitochondriotropic compounds such as methyl‐triphenyl‐phosphonium cation, triphenylphosphonium (TPP), dequalinium (DQA), rhodamine123, and (E)−4‐(1H‐indol‐3‐ylvinyl)‐*N*‐methylpyridiniumiodide.^[^ ^34^ ^]^

As highlighted in literature, the two physicochemical properties nanoparticles, i) size and ii) surface charge, are critical in the internalization process.^[^ ^35^, ^36^ ^]^ Smart design by considering the size and surface charge of a diverse array of materials conjugated with lipophilic cations has led to be used successfully as mitochondrial‐targeting nanocarriers, including liposomes,^[^ ^37^, ^38^, ^39^, ^40^ ^]^ micelles,^[^ ^41^, ^42^ ^]^ dendrimers,^[^ ^43^ ^]^ polymeric nanocarriers,^[^ ^44^ ^]^ and protein‐based nanoparticles.^[^ ^45^ ^]^ In this study, the size of Pdots is considered as small size particles < 10 nm, and they may be mostly internalized via passive diffusion as compared to larger size particles > 50–500 nm that are subject to the endocytosis processes. Beside the small size of Pdots, the positive surface potential plays pivotal roles in the onset of internalization and post‐internalization localization. The substantial effect of surface charge exemplified with MitoPorter liposomes with *ζ*‐potential +50 mV,^[^ ^46^ ^]^ as well as dual‐function (DF)‐MITO‐Porter (150 nm; *ζ*‐potential +30 mV) encapsulating macromolecules,^[^ ^47^ ^]^ are localized to the mitochondria via electrostatic interactions. On the other hand, the lower surface charge lipid–polymer hybrid nanoparticles (*ζ*‐potential +17.2 mV) have also exhibited mitochondria targeting^[^ ^48^ ^]^ that assures the importance of electrostatic interplay between cationic charge and negative surface potential of mitochondria. A comprehensive study showed that the localization of lipid‐PEG‐TPP functionalized Ceria‐nanoparticles (*ζ*‐potential +45 mV) and oleylamine‐lipid‐PEG functionalized Ceria nanoparticles (*ζ*‐potential −23 mV) in mitochondria and intracellular space, respectively.^[^ ^49^ ^]^ Our described NIR emissive Pdots are designed to exploit the promising potential of lipophilic cation that may induce mitochondrial localization. The penetration behavior of the NIR emissive Pdots in all tumor models is studied via fluorescence imaging. Findings show that Pdots effectively penetrate tumor structures. Pdots particularly, which tend to penetrate the tumor's central region, provide an essential opportunity for imaging and suppressing tumors before they grow. Our study demonstrates the potential of using these ultrasmall Pdots in intratumoral imaging and encourages further research in the future.

## 2 Experimental Section

### 2.1 Materials

The experiments utilized chemicals purchased from different suppliers, including Alfa Aesar, Sigma–Aldrich, TCI, and Acros. The chemicals were used as they were received, without additional purification, unless specifically mentioned. Deionized water with a resistance of 18.2 MΩ·cm was used in all experiments to minimize the presence of impurities that might impact the findings. The ^1^H and ^13^C nuclear magnetic resonance (NMR) data were obtained using a VARIAN, 400 MHz spectrometer, a reliable instrument for obtaining information on the structure of the molecules.

### 2.2 Synthesis of 3‐Butoxy‐4‐Methylthiophene (BuMT)

**Scheme 2:**
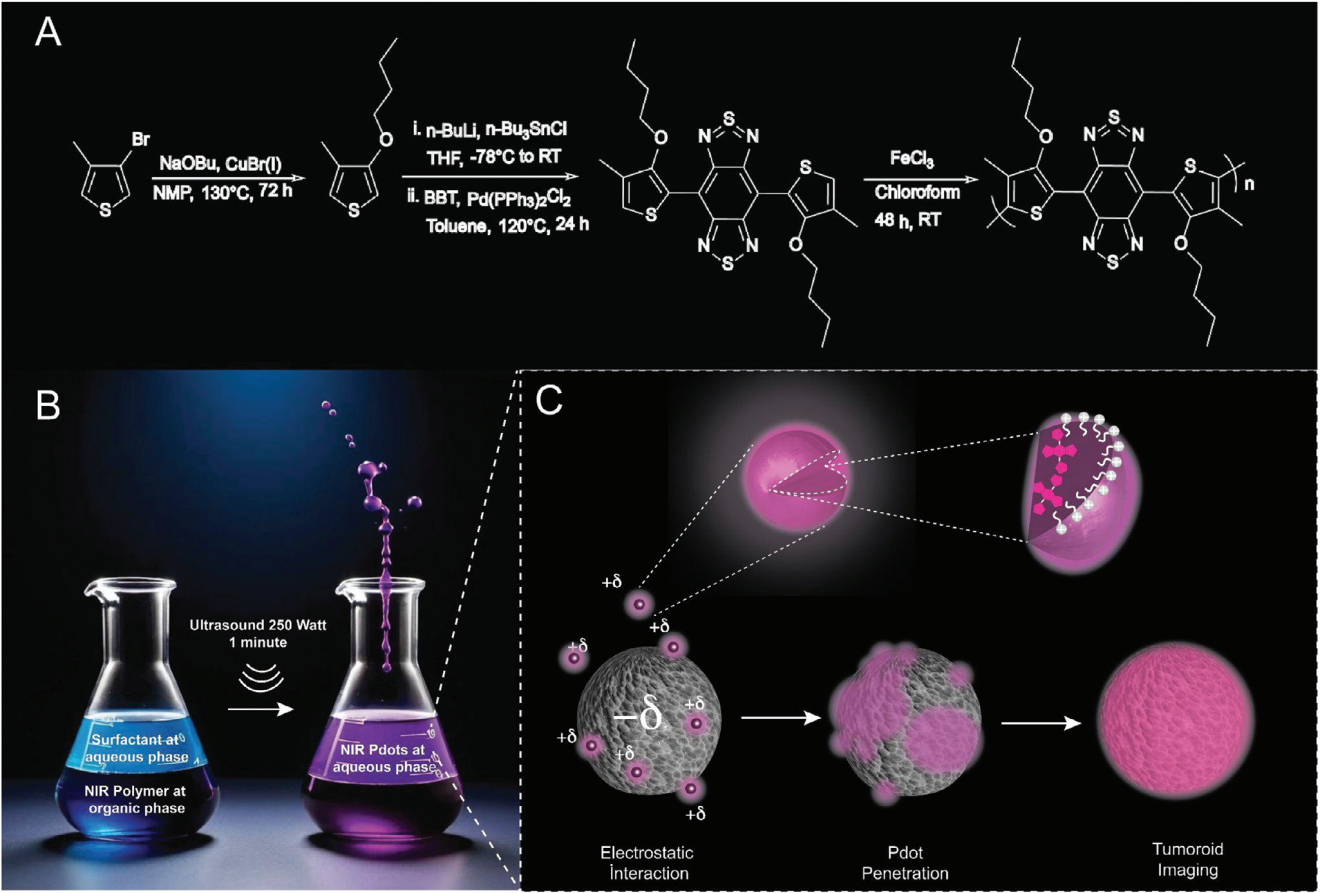
A) Synthesis protocol of NIR emissive Poly BT, B) schematic representation of the preparation of Pdots, and C) schematic illustration of the penetration behavior of the Pdot.

BuMT was synthesized as a donor source for the D–A–D monomer. Accordingly, 1.18 g of sodium metal was added into 20 mL of freshly distilled 1‐butanol, and after 1 h, a mixture of 25% by weight of NaOBu(Sodium butoxide)/1‐butanol was prepared under nitrogen gas. In the following step, 1‐methyl‐2‐pyrrolidone (0.5 mL, 5.20 mmol) and sodium butoxide in butanol (NaOBu) (25% by weight; 10.0 mL, 33.0 mmol) were added to a round bottom flask. After the mixture was heated to 130 °C, the reaction was continued by adding 3‐bromo‐4‐methylthiophene (BMT) (1 g, 5.6 mmol) and CuBr (0.8 g, 5.6 mmol). The reaction was carried out at 130 °C under a nitrogen atmosphere with a water‐cooled condenser (Reaction conditions are shown in **Scheme** **2**A). Then, the mixture was cooled to room temperature, and 10 mL of 0.25 g sodium bromide solution was added and stirred vigorously for 1 h. Next, the mixture was extracted with diethyl ether three times and filtered. The combined organic phase was washed twice with DI water and dried with MgSO_4_ (magnesium sulfate). The solvent was then removed using a rotary evaporator and purified by column chromatography (%100 Hexane). BuMT (3‐Butoxy‐4‐Methylthiophene) was obtained as a light‐yellow oil with a yield of 63% (0.60 g).

### 2.3 Synthesis of Monomer (4,8‐di‐3‐bromo‐4 Methylthiophenebenzobisthiadiazole)

D–A–D‐based monomer was synthesized with a Stille coupling reaction. BuMT(1.0 mmol) was prepared under cryoreaction conditions by dissolving with 10 mL of THF and cooling to −78 °C under an inert atmosphere. Next, butyl lithium (n‐BuLi, in 2.5 m hexane, 0.4 mL, 1.0 mmol) was added dropwise to the reaction medium. After stirring for 1.5 h at −78 °C, tributyltin chloride (0.33 g, 1.0 mmol) was added dropwise. At the end of this process, the reaction medium was allowed to reach room temperature slowly and mixed for 8 h at room conditions (Scheme 1A). At the end of the reaction, the product was extracted with ethyl acetate and washed at least twice with water. Afterward, it was dried with magnesium sulfate, and the solvent was evaporated with the rotary evaporator. After the first step, the stannylated product (1.0 mmol) and benzobisthiadiazole (BBT) (115 mg, 0.35 mmol) were dissolved in 10 mL of toluene, and Bis(triphenylphosphine)palladium chloride (Pd(PPh_3_)_2_Cl_2_) (78 mg) was added to the mixture. The mixture was stirred at 120 °C under a nitrogen atmosphere and in a water‐cooled condenser for 24 h (Scheme 1A). After completion of the reaction, it was cooled to room temperature extracted with ethyl acetate and washed at least two times with water. Subsequently, the solvent of the material dried with MgSO_4_, and the solvent was removed under vacuum. Afterward, the product was purified by column chromatography in a hexane/dichloromethane eluent (1:1) and stored at +4 °C ready to use. The monomer was obtained as dark blue, 90 mg in 51% yield.

### 2.4 Polymerization of Monomer

Polymerization of D–A–D monomer was performed to obtain the NIR emissive nonionic polymer via oxidative polymerization. A solution of monomer (8.4 mg, 0.016 mmol) in 2 mL of chloroform was added dropwise to a solution of anhydrous FeCl_3_ (15 mg, 0.09 mmol) in 2 mL of chloroform and stirred for 48 h at room temperature under a nitrogen atmosphere, as seen in Scheme 2A. After 2 days, the solvent of the mixture was evaporated, and the residue was washed with methanol for four times. Then, the polymer product was dried in a desiccator.

### 2.5 Preparation of Pdots

The miniemulsification process is schematically shown in Scheme 2B, which illustrates the formation of Pdots from poly BT (1 mg) dissolved in a water‐immiscible organic solvent (1 mL, CHCl_3_) in the presence of CTAB as a surfactant solution (84 mg surfactant in 10 mL MilliQ water). Upon 250‐Watt sonication for 2 min, turbid brownish solution was obtained. The sample was then stirred in a hot water bath to evaporate the organic solvent.

### 2.6 Atomic Force Microscopy (AFM) Imaging

In AFM imaging/analysis, CoreAFM from NanoSurf Inc. was used, and all results were evaluated by using NanoSurf CoreAFM software. To run the AFM/EFM (electrostatic force microscopy) imaging/analysis, a Multi 75E‐G type AFM tip was used for all three EFM analysis and topography imaging. During the EFM analysis, lifted tip scanning, also known as second scan forward, was performed with contour mode which provides path tracing of the first scan forward path in the second scan forward. To overcome secondary interactions between tip and substrate, a second scan forward height was selected as 135 nm at room temperature. All imaging and analysis were run via 512 point/line scanning, and each line scan time was adjusted to 1 s with no tip rotation in “Dynamic Scanning Mode”.

### 2.7 Pdot Localization in 2D Cell Culture and 3D Tumor Spheroid Imaging

SH‐SY5Y, MCF‐7, and PC‐12 cells were cultured in high glucose dulbecco's modified eagle medium (DMEM) (GIBCO, Thermo Fischer Scientific) containing l‐glutamine and supplemented with 10% Fetal Bovine Serum (GIBCO, Thermo Fischer Scientific) and 1% penicillin/streptomycin (GIBCO, Thermo Fischer Scientific). The cells were cultured to up to ≈90% confluency in a humidified environment (5% CO_2_, 37 °C). The harvested cell lines were seeded on 96‐well plates and incubated for 24 h. 190 nm Pdots were directly added to the cell culture medium and incubated for 24 h. After 24 h culturing, cells were analyzed under the fluorescence microscope (Zeiss Axio Observer 7). For the characterization of cells incubated with Pdot, the cell nucleus, cytoplasm, and mitochondria were labeled with 4′,6‐Diamidino‐2‐phenylindole (DAPI) (AAT Bioquest), Calcein Green (AAT Bioquest), and MitoView Green (BIOTIUM) respectively.

The cytotoxicity of Pdots on cells was investigated by Alamar Blue assay. The harvested cell lines were seeded on 96‐well plates and incubated for 24 h. Pdots were then applied to the cancer cells and incubated for 24 h. Later, Alamar Blue reagent was added to each well, incubated at 37 °C for 4 h, and measured at 570–600 nm using Multiskan GO Microplate Spectrophotometer (Thermo Fischer Scientific).

In 3D cell culture, spheroids were formed by the hanging drop method^[^ ^50^ ^]^ with the utilization of 25 × 10^3^ cells µL^−1^ cells for MCF‐7 and PC‐12, and 50 × 10^3^ cells µL^−1^ cells for SH‐SY5Y. 10 µL drops containing cells were dropped into the petri lids, and 2 mL of PBS was added to the petri dishes to maintain the humidity of the environment. Then, the petri lids were inverted, closed, and incubated for 24 h. Subsequently, the spheroids were moved to 24‐well plates and incubated for 24 h in a medium containing Pdot. After that, the spheroids were fixed using 4% paraformaldehyde (PFA), labeled with DAPI, MitoView Green, and Calcein Green, and visualized under fluorescence microscopy.

## 3 Results and Discussion

The NIR emissive conjugated polymer Poly BT was synthesized through a series of steps including substitution, still‐coupling reaction, and oxidative polymerization using FeCl_3_, as illustrated in Scheme 2A. This synthesis method provided a D–A–D type conjugation. The monomers BuMT and benzobisthiadiazole were chosen as the donor and acceptor, respectively. BuMT was characterized using ^1^H NMR and ^13^C NMR spectroscopy, with the corresponding spectra shown in Figures S1 and S2, respectively, confirming its structure. Mass spectroscopy (MS) showed that the BuMT had a mass of 170 g mol^−1^ and +1 proton of 171.0842 g mol^−1^ (Figure S3, Supporting Information), which means that the synthesis was successful. Subsequently, the D–A–D monomer (4,8‐di‐3‐bromo‐4 methylthiophenebenzobisthiadiazole) was synthesized and characterized using ^1^H NMR and ^13^C NMR spectroscopy. The ^1^H NMR spectra (Figure S4, Supporting Information) confirmed the proposed structure; while, the ^13^C NMR spectrum (Figure S5, Supporting Information) provided further validation. In addition, MS analysis gave a mass of 530 g mol^−1^ for the monomer, with a calculated +1 proton of 531.10 110 g mol^−1^ and 91.22% purity (Figure S6, Supporting Information), indicating successful synthesis with high purity. Optical characterization of the monomer (Figure S7, Supporting Information) revealed absorption and emission spectra with *λ*_max_ values of 616 and 810 nm, respectively.

The D–A–D monomer underwent polymerization via oxidative polymerization to yield the NIR emissive nonionic Poly BT, which was subsequently characterized using NMR spectroscopy. The ^1^H NMR spectrum of the resulting poly BT is depicted in Figure S8, Supporting Information, revealing notable changes such as peak broadening in the range of 1.0–2.0 ppm and the disappearance of aromatic hydrogens around 6.5–6.6 ppm, indicative of successful polymerization.

In Figure S9, Supporting Information, the optical characterization of poly BT is presented, demonstrating absorption and emission *λ*_max_ values of 660 and 860 nm, respectively. A significant red shift in comparison to the monomer (Figure S7, Supporting Information) and poly BT *λ*_max_ values is observed, corresponding to an increased conjugation length resulting from polymerization.

**Figure 1:**
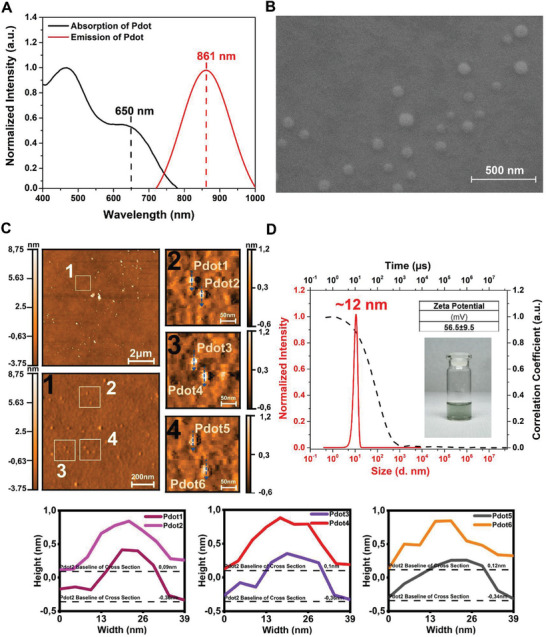
A) Absorption and emission spectra of NIR emissive Pdot in water. B) Scanning electron microscopy (SEM) image of NIR emissive Pdot (scale bar: 500 nm). C) Topography images and cross‐section analysis of the Pdots (Pdot1−6 bottom of this part) by AFM (scale bars for AFM images: 2 µm, 200 nm, and 50 nm, respectively). D) Size analysis, auto‐correlation graphs, and zeta potential value of NIR emissive Pdot by DLS with a photograph of the Pdot solution in water. Data are means ± SD, *n* = 3.

As we highlighted, Pdot was prepared from the nonionic Poly BT via the miniemulsion technique which homogenizes the size and surface charge. The Pdots consisted of a polymer composed of non‐ionic donor–acceptor–donor (DAD) polythiophene and CTAB cationic surfactant. The thiophene monomer 3‐butoxy‐4‐methyl thiophene was employed to provide solubility, and it was linked to the acceptor group benzobisthiadiazole via a single‐step Stille coupling reaction. The polythiophene was specifically engineered to emit light at wavelengths of 800 nm and beyond, ensuring coverage of the NIR region. The non‐ionic poly BT was effectively surrounded by the cationic charge added by CTAB, which did not affect its ability to emit. The illustration in Scheme 2B shows the proposed Pdot formation as depicting the organization of surface active molecules around nonionic polymers. The optical characterization was performed for the Pdot, and the absorption and emission maximum wavelengths were found as 650 and 861 nm in water (**Figure** **1**A). The absorbance maximum of 650 nm is attributed to the co‐planar geometry of the DAD structure, and 475 nm is mainly due to the twisted geometry of the polymer. NIR emission is mostly due to the photons absorbed by 650 nm. The emission peak of experimental spectra of Pdot was found to be within the range of 700–1000 nm, which corresponds to the NIR region, and the Stokes shift was calculated as 211 nm which minimizes self‐absorption of Pdot. As shown earlier, Pdots made of D–A–D type polymers were also promised large Stokes shifts and minimal self‐absorbance. The major advantage of NIR emissive Pdots was therefore obvious by their low interference with the emission signal.^[^ ^26^ ^]^ Moreover, the photostability of the NIR emissive Pdot was tested and the incident radiation intensity was calculated as 3.3 mV cm^−2^;^[^ ^51^ ^]^ the half‐life of the NIR emissive Pdot was found to be as 135.9 h (see Figure S10, Supporting Information).

The colloidal and morphological properties of Pdots were studied by SEM, and as seen in Figure 1B, spherical morphology was common for all Pdots. Further, AFM analysis was performed for the Pdot, and the average Pdot size was found as ≈20 nm (Figure 1C). Pdots’ cross‐sectional profiles (Pdot 1−6) prove the homogeneity in size and surface morphology. In addition to these, size analysis with dynamic light scattering (DLS, Figure 1D) yields the average hydrodynamic radius of the Pdot as 12 nm. The autocorrelation function shown in Figure 1D (dotted line) illustrates the monomodal relaxation that confirms the homogenous Pdot distribution. As suggested previously by Chui et. al., the fluorescence brightness of Pdots is mainly determined by the weight or volume fraction of conjugated polymer used in Pdot formation.^[^ ^52^, ^53^, ^54^ ^]^ As the previously described Pdot formation processes are not identical to the suggested process here, calculations are conducted based on the charge balance of CTAB micelles instead of the initial weight fraction (see the Supporting Information section for details). The fraction of NIR emissive polymers in Pdots is deduced to be 72% (Figure S11, Supporting Information), which suggests that semiconducting polymer content is in a moderately similar range as compared to earlier reports.^[^ ^52^, ^53^, ^54^ ^]^ In addition to these, an experiment is designed to reveal the number‐averaged of pure CTAB nanoparticles (NPs) and NIR emissive Pdots with the absence and the presence of nonionic polymer (all conditions such as initial concentration of surfactant, ultrasonication period, and energy are kept identical for both cases). The final sizes are measured for various trials and deduced as approximately ≈5 and ≈12 nm in the absence and the presence of nonionic polymer, respectively (Figure S12, Supporting Information). According to published research articles, the average hydrodynamic radius for CTAB NPs is found to be ≈2–5 nm.^[^ ^55^, ^56^, ^57^, ^58^ ^]^ Therefore, ≈5 nm size result was attributed to the CTAB NPs. On the other hand, the average hydrodynamic radius for NIR emissive Pdot was measured as nearly ≈12 nm. The difference between the sizes results was ascribed to the contribution of the NIR emissive polymers. As a result of the analysis, it was understood that most of the CTAB micelles include the NIR emissive polymer. The size difference between DLS and AFM analysis is attributed to the presence of and removal of solvents in these two analyses that cause a significant change in size for the latter case. The AFM and SEM results indicated that Pdots exhibit larger diameters than their solution diameter (DLS); this discrepancy is mainly due to the flattening of soft objects, which is induced by adherence and subsequent deformation. A similar observation has recently been discussed by Szebeni et al.: the diameter of lipid vesicle‐based particles determined via AFM was larger than their DLS results.^[^ ^59^ ^]^ The zeta potential of Pdot confirmed that non‐ionic Pdots were colloidally stabilized in water due to their surface charge (Zeta Potential = 56.5 ± 9.5 mV; see in Table S1, Supporting Information) provided by CTAB. The relatively high zeta potential above 40 mV had been evaluated as a colloidal stability criterion.^[^ ^60^ ^]^ In addition, the colloidal stability of Pdots at different time points (1., 15., and 30. Day) varying pH (between 5.5 and 7.5) had been tested and are presented in Table S2, Supporting Information, which shows the size, zeta potential (ZP), mobility (Mob), and conductivity (Cond) values. As shown in Table S2 and Figure S13, Supporting Information, pH has a slight effect on the size and zeta potential of Pdots, and it does not cause instability. In particular > 50 mV zeta potential values at all pH values confirm colloidal stability. A similar charge distribution is expected at the dried state, which is confirmed by electrostatic force microscopy (EFM), as shown in Figure S14, Supporting Information. The Pdot‐AFM tip interaction is negligible if the tip is at 0 V; however, once the tip is biased to the 3 V/−3 V, electrostatic interactions (attractive and repulsive forces) are dominant. The phase angle under 3 V and −3 V scanning is measured as 1.15° and 2.00°, respectively, which indicates particles are positively charged. This confirms that cationic Pdots are stabilized and remain intact upon drying. The argument that particles with a positive surface charge can penetrate the cell more efficiently is confirmed by cell culture experiments with different cancer cell lines (as shown in Scheme 2C, the surface charge of Pdots may induce mutual electrostatic interactions with tumor models).

**Figure 2:**
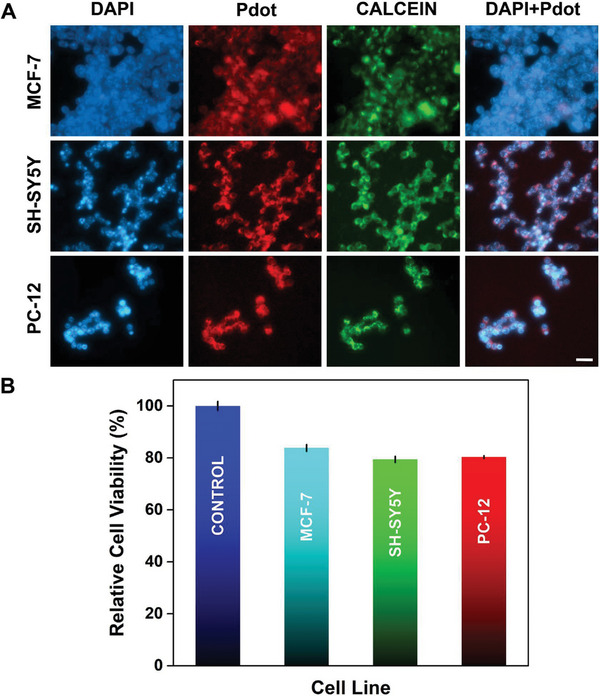
A) Fluorescence microscopy images of MCF‐7, SH‐SY5Y, and PC‐12 cells, cultured with Pdot and labeled with DAPI and Calcein Green (blue: DAPI, red: Pdot, green: Calcein, scale bar: 20 µm). B) Relative cell viability of cells cultured with Pdot obtained via Alamar blue assay (Pdot concentration: 190 nm) (*n* = 3, *p* < 0.0001).

The conventional 2D culture using MCF‐7, SH‐SY5Y, and PC‐12 cells was performed by incubating with Pdots. **Figure** **2**A shows normalized fluorescence images captured through Pdot and DAPI, respectively; the staining of nuclei was achieved for all cell lines. As explained earlier, cationic Pdots exhibit a strong tendency to penetrate the nucleus^[^ ^28^, ^29^ ^]^ of cancer cells whereas they accumulate mostly in the cytoplasm of healthy cells. As seen in Figure 2A, the Pdot and DAPI signals overlap perfectly with each other for the three cancer cell lines on the right column merge images. The cytotoxicity of Pdot was analyzed with Calcein Green staining, confirming the cell viability in the presence of Pdots. As clearly shown in Figure 2A, high cell viability was observed while Pdots penetrated the cells successfully. To support these findings, an Alamar Blue cell viability assay was also performed, confirming the high cell viability of cells after Pdot incubation, as shown in Figure 2B. After incubation with Pdot, MCF‐7 cells showed 84% viability, SH‐SY5Y cells 79%, and PC‐12 cells 80%. Moreover, the staining of mitochondria was achieved for all cell lines after incubation with Pdot (Figure S15, Supporting Information). It was observed that the Pdot and MitoView signals overlapped perfectly with each other for the three cancer cell lines (Figure S15, Supporting Information; merge image on the right column). These findings assure that cationic Pdots hold promise in divergent modalities for nuclei imaging as well as the allocation of organelles. The cells were cultured with Pdots for 3, 5, and 7 days and analyzed via a fluorescence microscope. Figures S16–S18, Supporting Information show Pdot, DAPI, and Calcein Green staining images on days 3, 5, and 7. It has been noted that extended incubation time caused no decay, and Pdots maintained their photostability. In addition, it was also noted that after long‐term cell culture, Pdots still exhibited a strong tendency to penetrate to the nucleus. Besides, Calcein Green staining confirmed the high cell viability for long‐term incubation in the presence of Pdots.

**Figure 3:**
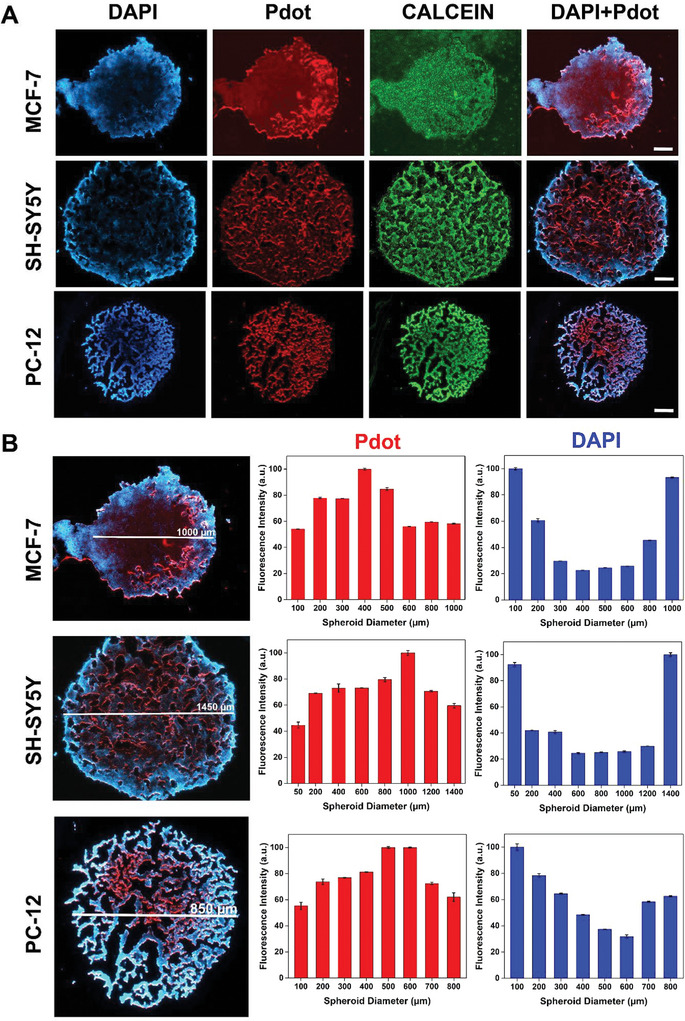
A) Fluorescence microscopy images of 3D MCF‐7, SH‐SY5Y, and PC‐12 cell spheroids cultured with Pdot and labeled with DAPI and Calcein Green (blue: DAPI, red: Pdot, green: Calcein, scale bar: 200 µm). B) Fluorescence intensity measurement of Pdot and DAPI diametrically for 3D spheroids (*n* = 3, *p* < 0.0001).

3D cell culture was performed using hanging drop methodology with MCF‐7, SH‐SY5Y, and PC‐12 cells (Figures S19–S21, Supporting Information), and the circularity was calculated as 0.86, 0.76, and 0.83, respectively (Figure S22, Supporting Information). The circularity is a significant characteristic for 3D spheroids and if the value is close to 1, it indicates that the spheroid has an ideally circular structure.^[^ ^61^ ^]^ After that, the 3D tumor spheroids were incubated with Pdot. Further, 3D spheroid imaging was achieved using DAPI, Pdot, and Calcein Green as shown in **Figure** **3**A. As seen in Figure 3A, DAPI localized more densely at the outer periphery of the spheroid but weakened toward the center of the spheroid. On the contrary of the DAPI, Pdot showed that it could intensely penetrate the center of the spheroid (Figure 3A merge image on the right column). In addition, cell viability was demonstrated with Calcein Green staining after incubation with Pdot. Moreover, Figure 3B shows the fluorescent intensity measurement of Pdot and DAPI diametrically for MCF‐7, SH‐SY5Y, and PC‐12 respectively. Unlike DAPI, toward the center of the spheroid, the Pdot intensity increased and could penetrate further into the spheroids. The previous studies suggest that the zeta potential of tumoroids is a major driving force in the internalization or translocation of nanoparticles; for example, the use of cationized PEI‐based nucleic acid delivery systems was suggested by their cationic charges.^[^ ^62^, ^63^, ^64^, ^65^, ^66^ ^]^ The Pdots described in this work exhibited 56.5 ± 9.5 mV which generated large potential differences between tumoroids. This showed that Pdot could penetrate into compact 3D tumor spheroids and have the potential to be used in imaging.

**Figure 4:**
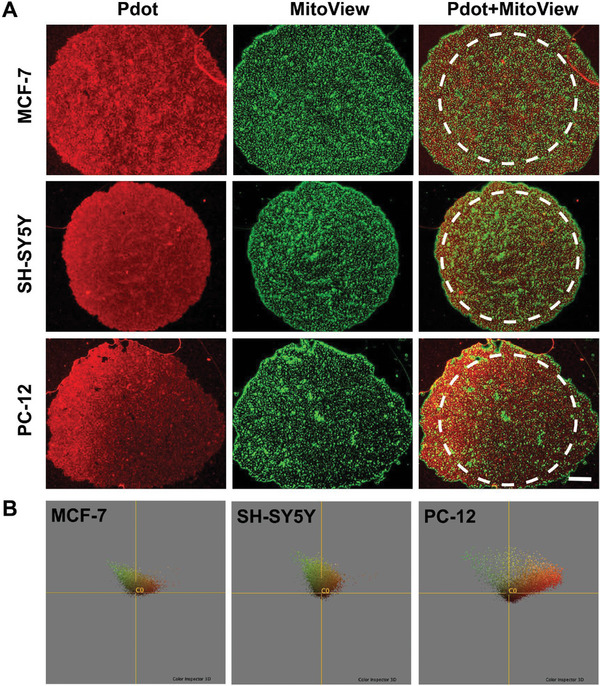
A) Fluorescence microscopy images of 3D MCF‐7, SH‐SY5Y, and PC‐12 cell spheroids cultured with Pdot and labeled with MitoView Green (red: Pdot, green: MitoView, scale bar: 200 µm). B) Pdot distribution and colocalization of Mitoview analysis with KLT/PCA for MCF‐7, SH‐SY5Y, and PC‐12 cell spheroids.

After incubation with Pdot, 3D spheroids were subjected to visualization using MitoView Green, a fluorescent dye recognized for its specificity in staining mitochondria, as depicted in **Figure** **4**A. To date, mitochondria‐targeted probes have been created. These probes efficiently penetrate through biological membranes and muscle cells, preventing mitochondrial damage and potentially serving as the foundation for mitochondria‐protective therapeutics. That's why, we also target the mitochondria to investigate the potential of our NIR emissive Pdot as an organelle‐targeted therapeutics. In addition, mitochondria are regarded as one of the most effective cancer therapeutic targets due to their significance in oxidative metabolism and apoptosis. As a result, mitochondrial targeting offers alternate possibilities for cancer therapy that targets mitochondria.^[^ ^67^, ^68^, ^69^, ^70^, ^71^, ^72^ ^]^ Our observations unveiled intriguing co‐localization patterns of Pdot and MitoView Green within distinct regions of the spheroids, indicative of the potential translocation of Pdot into the mitochondria. This phenomenon aligned with prior studies, suggesting the ability of certain nanoparticle formulations, such as quantum dots and carbon dots, to traverse cellular membranes and localize within subcellular organelles, including mitochondria, owing to their size and surface properties.^[^ ^73^, ^74^, ^75^ ^]^ However, it's essential to acknowledge that the presence of Pdot staining in regions does not fully overlap with MitoView Green staining, implying interactions with cellular components beyond mitochondria. These interactions may involve membrane binding, cytoplasmic distribution, or association with other cellular structures. Understanding the molecular mechanisms underlying such interactions warrants further investigation and may provide insights into the cellular uptake and intracellular trafficking pathways of Pdots. Therefore, the distribution as well as colocalization of Mitoview and Pdots were analyzed via Karhunen–Loeve transform (KLT) and principal components analysis (PCA), with the aid of software FIJI, as shown in Figure 4B (analysis details given in Figure S23, Supporting Information). Here, the red channel (Pdots) and green channel (MitoView) of overlaid images were processed fixed number of pixels. The interpretation of the KLT/PCA analysis results indicating differences in distribution between PC‐12, SH‐SY5Y cell‐based and MCF‐7 cell‐based tumor spheroids can provide valuable insights into the characteristics of these cell lines and their interactions with the Pdots and MitoView stain. The data points being closer to the red axis in the KLT/PCA color space suggest that the variations in the dataset are more aligned with the direction represented by the red channel (Pdots). This alignment could imply a higher intensity or presence of features captured by the red channel, indicating a stronger association or interaction between the Pdots and the PC‐12 cell‐based spheroids. The red‐stained features may correspond to specific cellular structures or components within the PC‐12 spheroids, such as cytoplasmic regions or membrane‐associated proteins, which exhibit distinct fluorescence characteristics with the Pdots. The clustering of data points along the red axis may reflect consistent patterns or distributions of these features across the PC‐12 spheroids, suggesting a more uniform response to the Pdots treatment. In contrast, the accumulation of data points closer to the origin in the KLT/PCA color space for SH‐SY5Y and MCF‐7 cell‐based spheroids indicates a more dispersed distribution with less pronounced directional alignment. This distribution suggests that the variations in the dataset are not strongly aligned with any particular color channel, indicating a more balanced contribution from all color channels (red, green, and possibly blue). The accumulation of data points near the origin may suggest a more heterogeneous response to the Pdots and MitoView stains within the SH‐SY5Y and MCF‐7 spheroids, with a wider range of fluorescence intensities and patterns across different regions of the spheroids. The SH‐SY5Y and MCF‐7 spheroids may exhibit varied cellular morphology, proliferation rates, or metabolic activities compared to PC‐12 spheroids, resulting in a more diverse and dispersed distribution of fluorescence signals in the KLT/PCA color space.

In summary, the differences in the distribution of data points between PC‐12 and SH‐SY5Y MCF‐7 cell‐based tumor spheroids in the KLT/PCA color space reflect distinct interactions between the cells and the Pdots and Mito View, as well as potential differences in cellular characteristics and behavior. Here, it may be noted that the colloidal stability and high surface charge (> +40 mV) of cationic Pdots are crucial determinants of their cellular uptake and subcellular localization. The positive surface charge facilitates electrostatic interactions with negatively charged cell membranes, promoting cellular internalization via endocytosis pathways. In addition, the surface charge plays a pivotal role in determining the intracellular fate of nanoparticles, influencing their interactions with intracellular components and subcellular organelles.^[^ ^69^, ^70^, ^71^, ^72^, ^76^ ^]^ Overall, our findings contribute to the growing body of evidence supporting the multifaceted applications of cationic Pdots in biomedical imaging and highlight their potential for elucidating complex cellular processes in both physiological and pathological contexts. Further research exploring the mechanistic basis of Pdot‐cell interactions and optimizing their physicochemical properties will undoubtedly enhance their efficacy and versatility in biomedical research and clinical applications.

Finally, the ability of Pdots to penetrate tumor spheroids is influenced by several factors, including i) size, ii) surface charge, and iii) surface chemistry and mechanical properties. In the context of soft cationic nanodots, their unique physicochemical characteristics may confer advantages in Pdots, particularly those with soft and deformable structures, and may demonstrate higher cellular internalization efficiency compared to negatively charged nanoparticles. The cationic and neutral particles were proven to be effectively penetrable as compared to negatively charged particles, and earlier investigations have determined that cationic and neutral particles exhibit superior transport efficiency when contrasted with negatively charged particles. As stated previously, this advantage stems from the attraction between the positive nanoparticles (NPs) and the negatively charged surface of the cell membrane, thereby augmenting both the speed and extent of internalization.^[^ ^29^, ^77^, ^78^, ^79^, ^80^, ^81^, ^82^ ^]^ This increased internalization efficiency can be attributed to the favorable interactions between Pdots and cell membranes, as well as to the ability of soft nanoparticles to undergo shape transformation or membrane fusion processes, facilitating their uptake by tumor cells.

## 4 Conclusion 

This study describes the formation of single‐chain Pdots via ultrasonic emulsification of nonionic D–A–D type Poly BT with amphiphilic CTAB. The methodology yields Pdots with a high cationic surface charge (+56.5 mV ± 9.5) and an average hydrodynamic radius of 12 nm. Optical characterization reveals that these Pdots emit near‐infrared (NIR) light at a maximum wavelength of 860 nm. The significant advantage of positively charged Pdots is demonstrated in diffusion‐limited mediums such as tissues, utilizing MCF‐7, SH‐SY5Y, and PC‐12 tumor spheroid models. Fluorescence microscopy analysis of tumor spheroids from MCF‐7, SH‐SY5Y, and PC‐12 cell lines reveals that the intensity profile of Pdots confirming extensive penetration into the central regions of the models. The ability of Pdots to penetrate tumor spheroids is influenced by several factors, including i) size, ii) surface charge, and iii) surface chemistry and mechanical properties. In the context of soft cationic nanodots, their unique physicochemical characteristics may confer advantages in Pdots, particularly in those with soft and deformable structures, and may demonstrate higher cellular internalization efficiency compared to other nanoparticles. Notably, mitochondria staining dye reveals an overlap between the regions stained by Pdots and the dye in all three tumor spheroid models. Our results suggest that Pdots exhibit a long‐range mean free path of penetration (≈1 µm) in dense mediums and tumors. The analysis suggests that PC‐12 and SH‐SY5Y MCF‐7 cell‐based tumor spheroids in the KLT/PCA color space reflect distinct interactions between the cells and the Pdots and Mito View, as well as potential differences in cellular characteristics and behavior. Here, it may be noted that the colloidal stability and high surface charge (> +40 mV) of cationic Pdots are crucial determinants of their cellular uptake and subcellular localization. Overall, the soft nature and positive surface charge of Pdots confer distinct advantages in penetrating tumor spheroids compared to negatively charged nanoparticles. By exploiting their unique physicochemical properties, Pdots hold promise for enhancing the delivery and distribution of therapeutic agents within solid tumors, ultimately improving the efficacy of cancer treatment strategies. Further elucidation of the mechanisms underlying the penetration of Pdots into tumor spheroids will contribute to the development of more efficient nanomedicine platforms for cancer therapy.

## 5 Statistical Analysis

Unless otherwise indicated, each statistical analysis had a sample size (*n*) of at least three. Every error bar displayed is the mean ± SD. The figure legends include *p‐*values and all statistical analyses. The statistical analysis was performed using Excel and GraphPad Prism 9 software (GraphPad Prism, Inc., San Diego, USA), which employed one‐way ANOVA.

## Conflict of Interest

The authors declare no conflict of interest.

## Author Contributions

The manuscript was written through the contributions of all authors. All authors have approved the final version of the manuscript.

## Supporting information

Supporting Information

## Data Availability

The data that support the findings of this study are available from the corresponding author upon reasonable request.
