# Supplementary material for: Near‐Infrared Emissive Super Penetrating Conjugated Polymer Dots for Intratumoral Imaging in 3D Tumor Spheroid Models
Source: Adv Sci (Weinh). 2024 Jul 18;11(35):2403398. doi: 10.1002/advs.202403398 (PMC11425279; doi:10.1002/advs.202403398)
Supplement: Supplementary file 1 — Supporting Information [file ADVS-11-2403398-s001.docx]

**Electronic Supplementary Information**

Near Infrared Emissive Super Penetrating Conjugated Polymer Dots for Intratumoral Imaging in 3D Tumor Spheroid Models

**Soner Karabacak^a^, Başak Çoban^b^, Ahu Arslan Yıldız^b^, Ümit Hakan Yıldız^a^***

^a^ Department of Chemistry, Izmir Institute of Technology, Urla, 35430, Izmir, Türkiye

^b^ Department of Bioengineering, Izmir Institute of Technology, Urla, 35430, Izmir, Türkiye

E−mail: [hakanyildiz@iyte.edu.tr](mailto:hakanyildiz@iyte.edu.tr)

**Table of Contents**

|  | Page Number |
| --- | --- |
| Figure S1. ^1^H NMR spectrum of BuMT. | **S4** |
| Figure S2. ^13^C NMR spectrum of BuMT. | **S5** |
| Figure S3. Mass spectrum of BuMT | **S6** |
| Figure S4. ^1^H NMR spectrum of Monomer. | **S7** |
| Figure S5. ^13^C NMR spectrum of Monomer. | **S8** |
| Figure S6. Mass spectrum of monomer. | **S9** |
| Figure S7. Absorption and emission spectra of D−A−D monomer | **S10** |
| Figure S8. Stack ^1^H NMR spectrum of Poly BT | **S11** |
| Figure S9. Absorption and Emission spectra of NIR emissive Poly BT | **S12** |
| Figure S10. The graph of ln(A_t_/A_0_) versus time for the NIR emissive Pdot | **S13** |
| Figure S11. Analysis of Pdots Zeta Potential | **S14** |
| Figure S12. Number-averaged size measurements of CTAB and Pdots | **S15** |
| Figure S13. Time and pH dependent size analysis | **S16** |
| Figure S14. EFM Topography Images of Pdot | **S17** |
| Figure S15. Fluo. mic. images of MCF−7, SH−SY5Y, and PC−12 cells, cultured with Pdot and labeled with DAPI and MitoView | **S17** |
| Figure S16. Fluorescence microscopy images of MCF−7 cells cultured with Pdot for 3, 5, and 7 days labeled with DAPI and Calcein Green | **S18** |
| Figure S17. Fluorescence microscopy images of SH−SY5Y cells cultured with Pdot for 3, 5, and 7 days labeled with DAPI and Calcein Green | **S19** |
| Figure S18. Fluorescence microscopy images of PC−12 cells cultured with Pdot for 3, 5, and 7 days labeled with DAPI and Calcein Green | **S20** |
| Figure S19. Light microscopy images of MCF−7 cell spheroids were obtained in 24 h via hanging drop methodology | **S21** |
| Figure S20. Light microscopy images of SH−SY5Y cell spheroids were obtained in 24 h via hanging drop methodology | **S21** |
| Figure S21. Light microscopy images of PC−12 cell spheroids were obtained in 24 h via hanging drop methodology (scale bar: 200 µm). | **S22** |
| Figure S22. Circularity analysis of 3D cell spheroids obtained via hanging drop methodology. | **S22** |
| Figure S23. Screen shots of the FIJI Color Inspector 3D and Karhunen−Loève Transform (KLT) analysis cell spheroids. | **S24** |


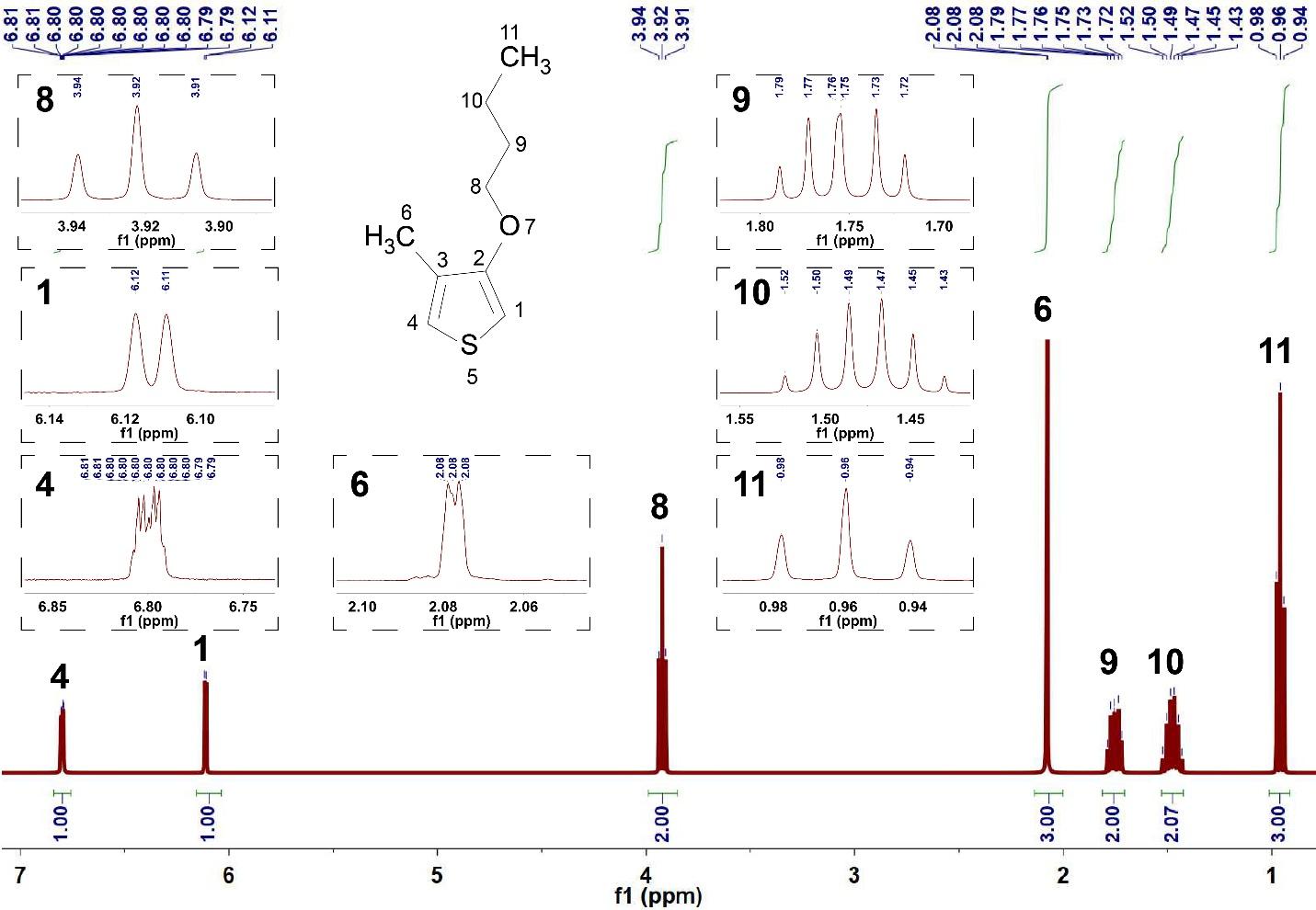


**Figure S1.** ^1^H NMR spectrum of BuMT. ^1^H NMR (400 MHz, CDCl_3_) δ 6.81 – 6.79 (m, 1H), 6.11 (d, J = 3.2 Hz, 1H), 3.92 (t, J = 6.4 Hz, 2H), 2.08 (s, 3H), 1.79 – 1.72 (m, 2H), 1.52 – 1.43 (m, 2H), 0.96 (t, J = 7.4 Hz, 3H).


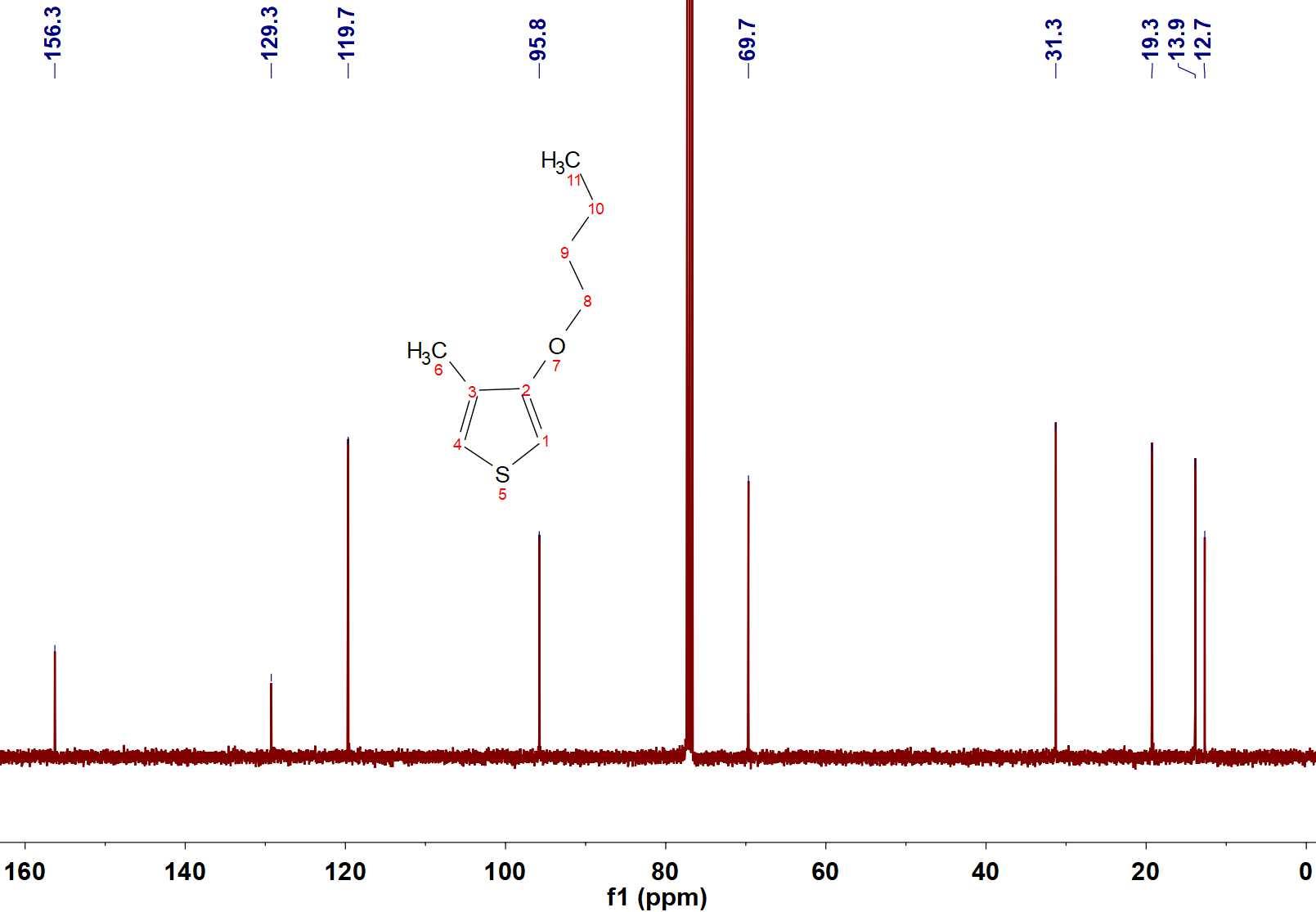


**Figure S2**. ^13^C NMR spectrum of BuMT. ^13^C NMR (101 MHz, CDCl_3_) δ 156.3, 129.3, 119.7, 95.8, 69.7, 31.3, 19.3, 13.9, 12.7.

Chromatographic separation was performed using a HPLC Agilent 1260 Infinity series (Agilent Technologies, Santa Clara, CA, USA) instrument with a Poroshell 120 EC-C18 (3.0X50 mm, 2.7 µm particle size) column. The mobile phase system was constructed using a gradient elution of 0.1% formic acid in water (A) and acetonitrile (B) as follows: 0-0.5 minutes, 10% B; 0.5-5 min, 70% B; 5-7 min, 95% B; 7-10 min, 95% B; 10-15 min, 10% B. The column oven was maintained at 35 °C. The injected sample volume was 10 µL and the flow rate used was selected as 0.5 mL/min.

MS analysis was performed using an Agilent 6550 iFunnel high resolution Accurate Mass Q-TOF/MS equipped with the Agilent Dual Jet Stream electrospray ionization (Dual AJS ESI) interface operating in positive ion in the following cases: drying gas flow, 14.0 L/min; nebulizer pressure, 35 psi; gas drying temperature, 290 °C; sheath gas temperature, 400 °C; sheath gas flow, nitrogen 12 L/min. MS/MS spectra were collected with collision energies of 10 eV.  The scanning range m/z was selected from 40 to 400. The acquisition was controlled by Agilent MassHunter Acquisition Software Ver. A.09.00 and the data were processed with MassHunter Qualitative Software Ver. B.07.00.


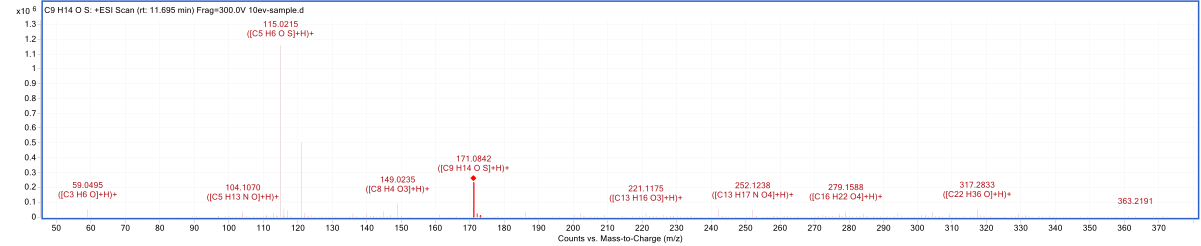


**Figure S3**. Mass spectrum of BuMT (Molecular ion of (BuMT+1)).


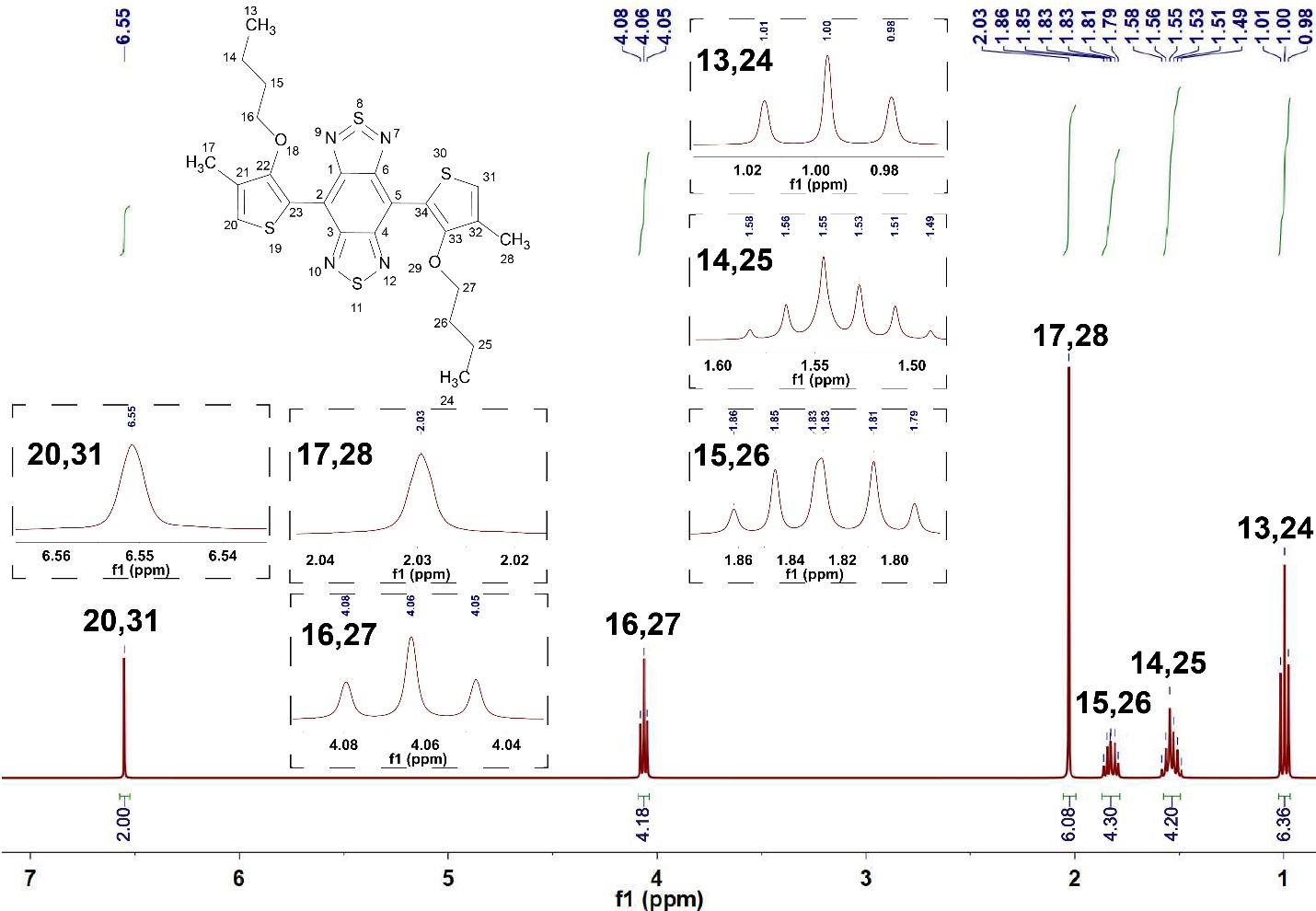


**Figure S4**. ^1^H NMR spectrum of Monomer. ^1^H NMR (400 MHz, CDCl_3_) δ 6.55 (s, 2H), 4.06 (t, J = 6.4 Hz, 4H), 2.03 (s, 6H), 1.86 – 1.79 (m, 4H), 1.58 – 1.49 (m, 4H), 1.00 (t, J = 7.4 Hz, 6H).


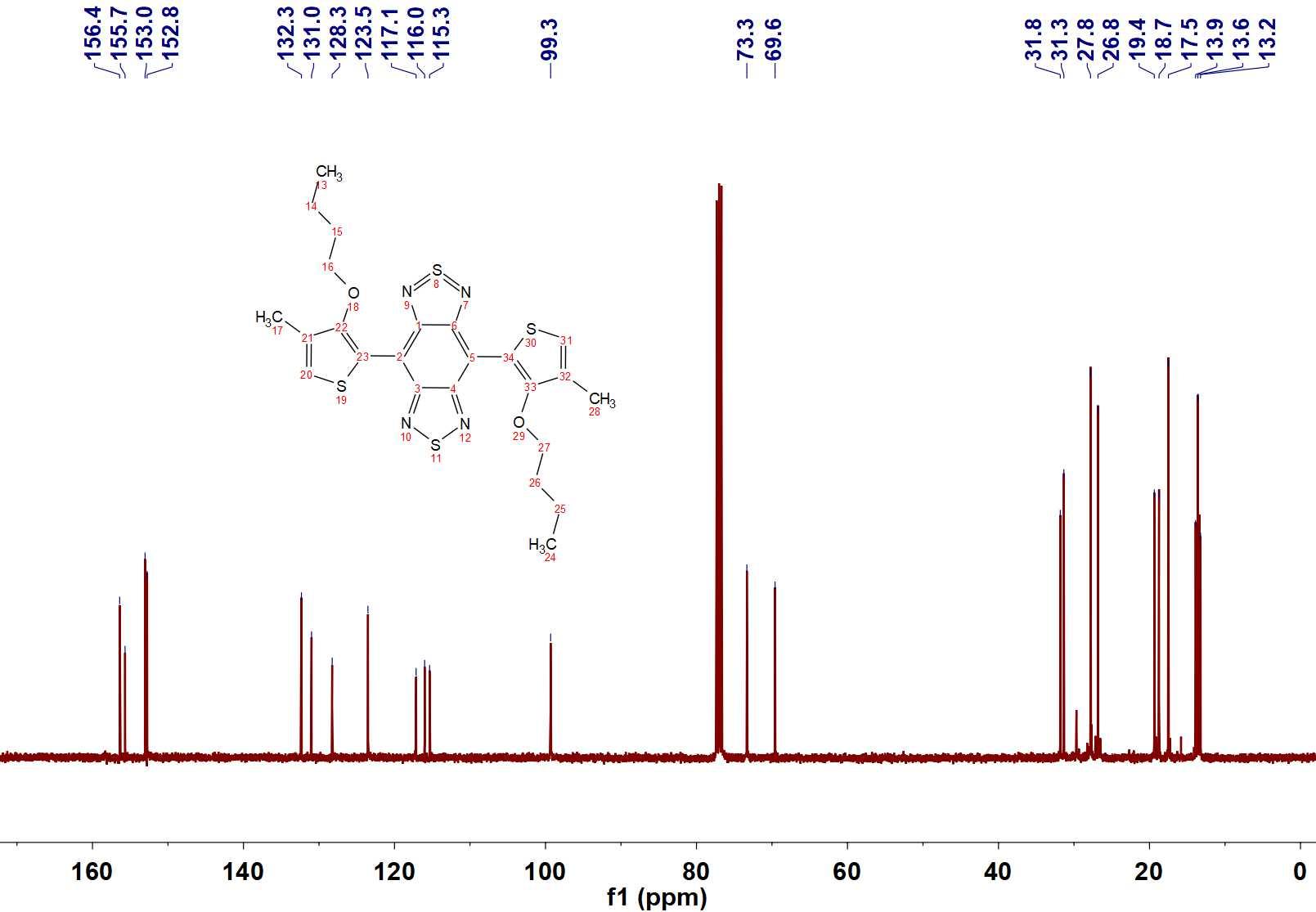


**Figure S5**. ^13^C NMR spectrum of Monomer. ^13^C NMR (101 MHz, CDCl_3_) δ 156.4, 155.7, 153.0, 152.8, 132.3, 131.0, 128.3, 123.5, 117.1, 116.0, 115.3, 99.3, 73.3, 69.6, 31.8, 31.3, 27.8, 26.8, 19.4, 18.7, 17.5, 13.9, 13.6, 13.2.


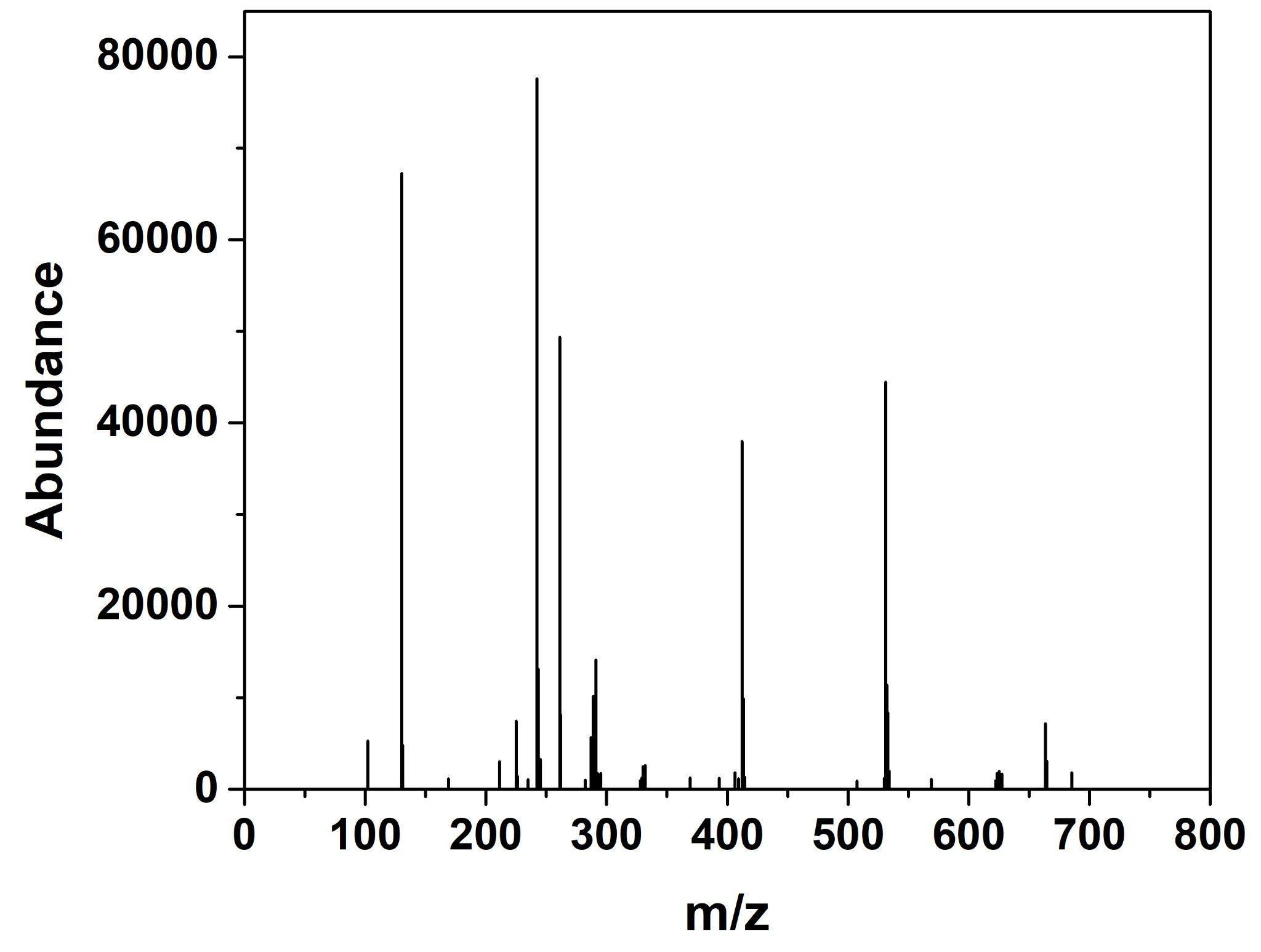


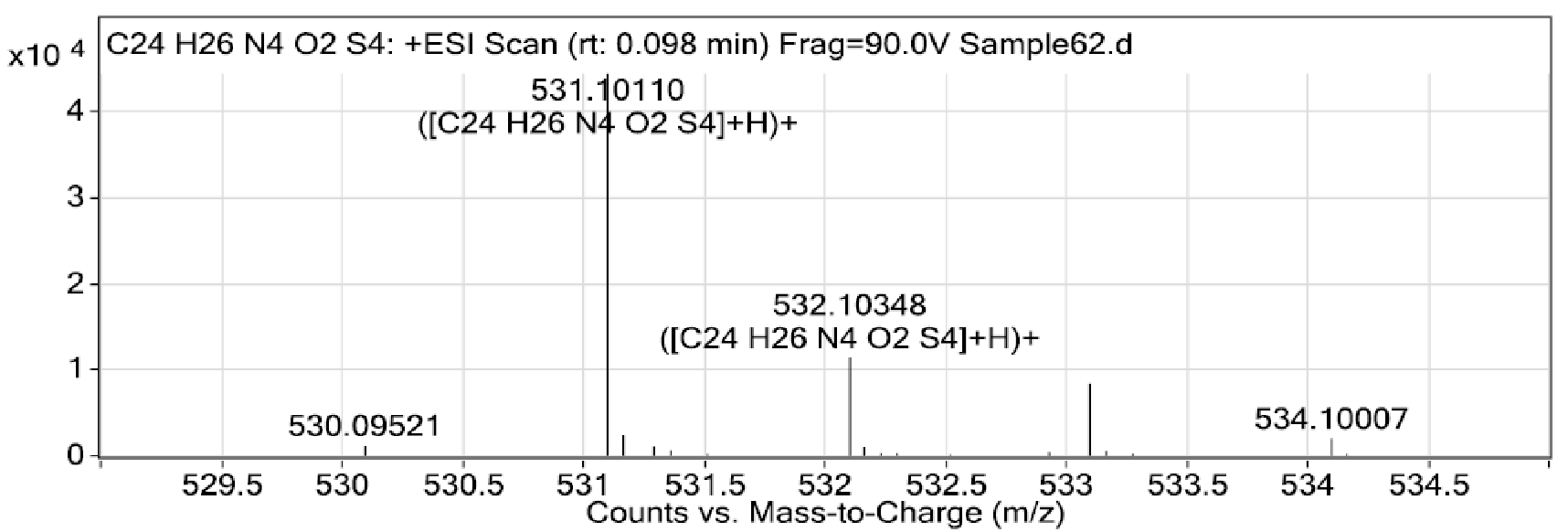


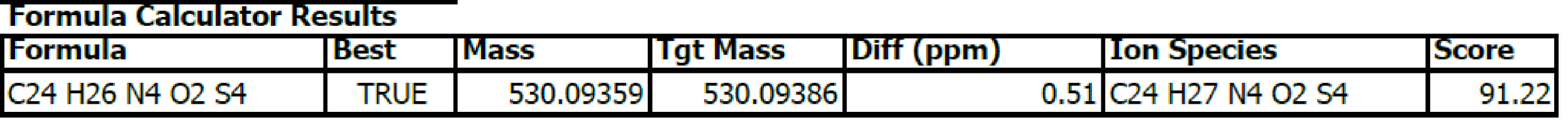


**Figure S6**. Mass spectrum of monomer.


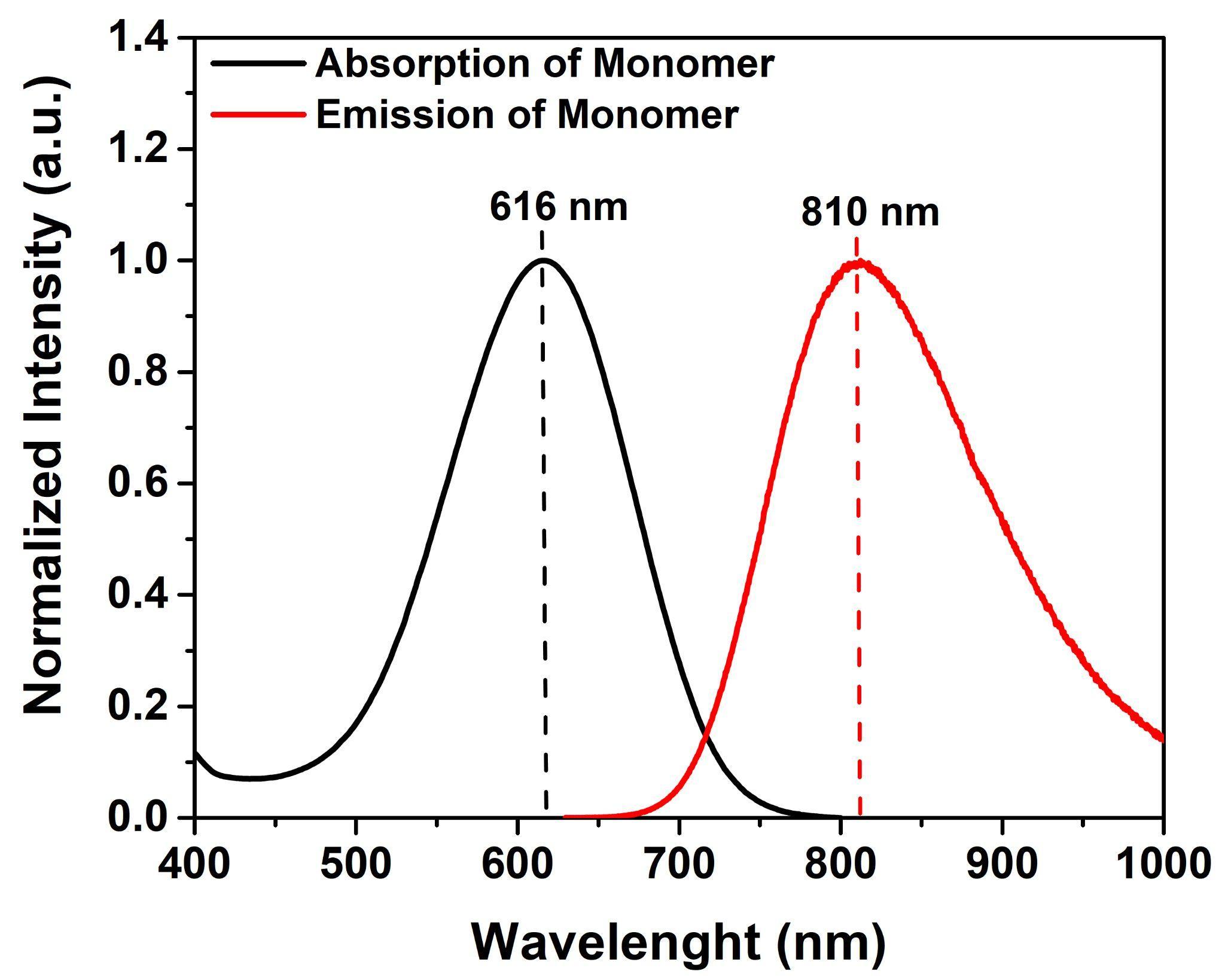


**Figure S7**. Absorption and emission spectra of D−A−D monomer in chloroform.

**
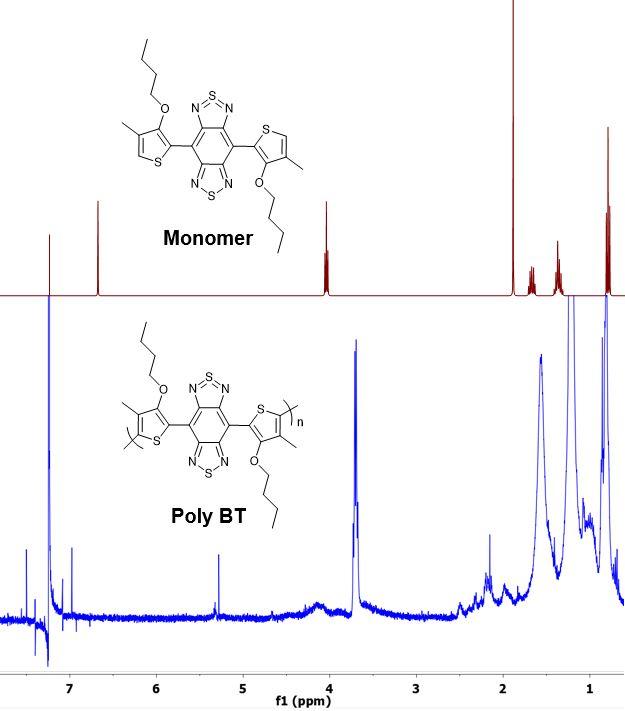
**

**Figure S8**. Stack ^1^H NMR spectrum of NIR emissive Poly BT with its monomer.


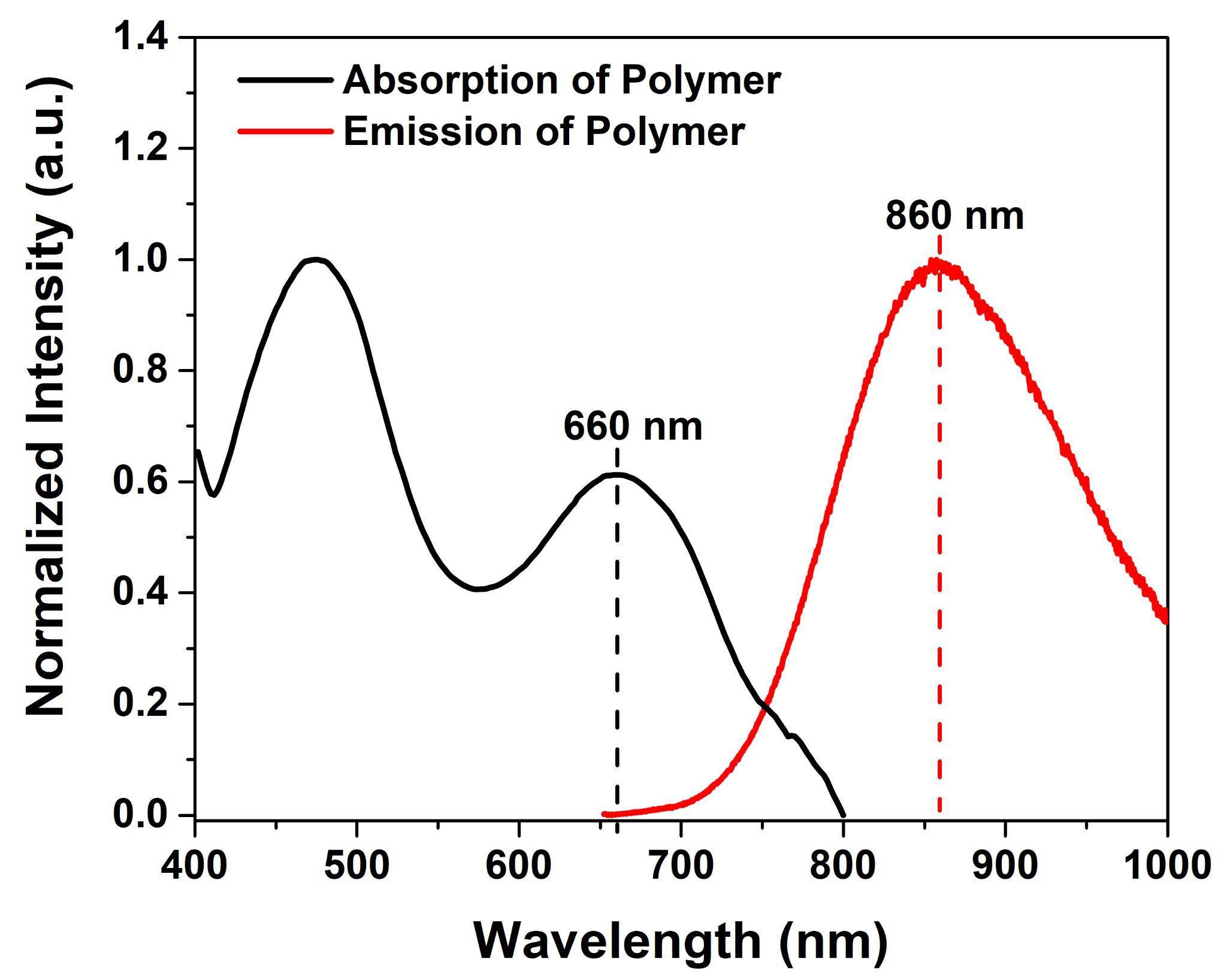


**Figure S9**. Absorption and Emission spectra of NIR emissive Poly BT in chloroform.

The Pdot sample was placed in a glass vial. and the sample was positioned 15 cm away from the light source (red LED, 620–630 nm). Absorbance measurements were performed for 5 hours at 30−minute time intervals, and the degradation rate was calculated from the equation (ln(A_t_/A_0_) = k_deg_ x t) (Figure S10).


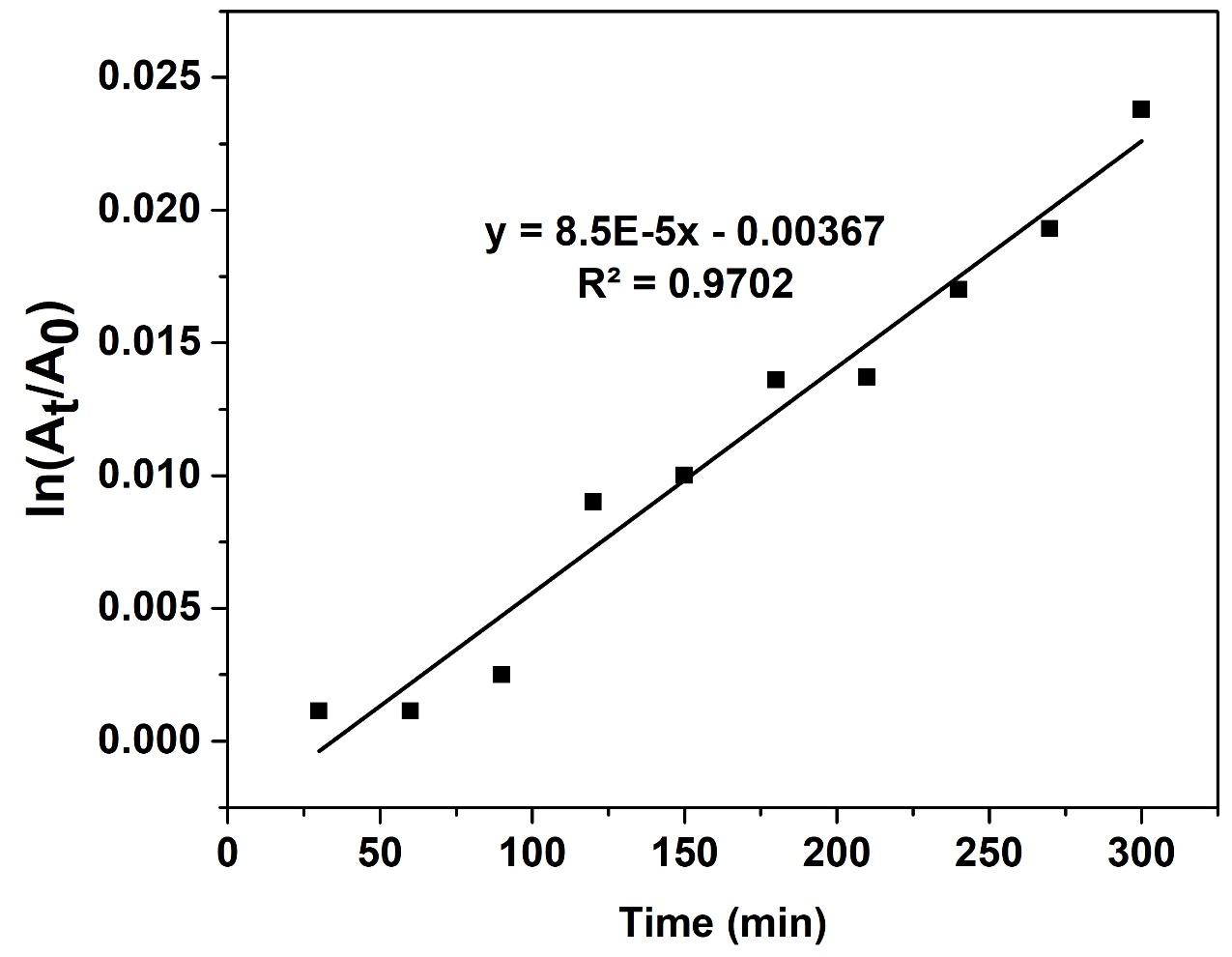


**Figure S10**. The graph of ln(A_t_/A_0_) versus time for the NIR emissive Pdot.

**Table S1**. Zeta potential measurement for NIR emissive Pdot.

|  | **ZP** | **Mob** | **Cond** |
| --- | --- | --- | --- |
|  | **(mV)** | **(µmcm/Vs)** | **(mS/cm)** |
| **NIR emissive Pdot** | 56.5±9.5 | 4.43±0.75 | 0.191 |


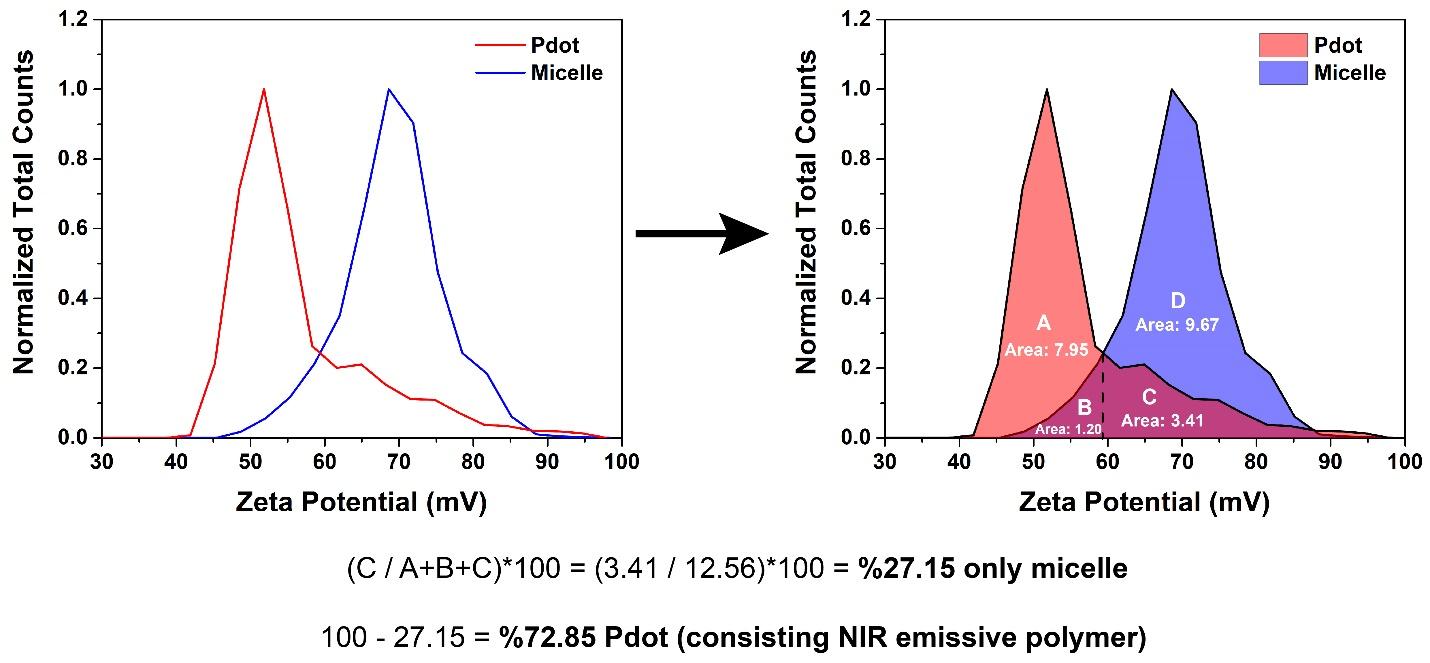


**Figure S11**. Analysis of Pdots containing NIR emissive polymers in solution with Zeta potential measurements.

The experiment was designed to deduce the zeta potential of CTAB vesicles in the absence and the presence of nonionic polymer (all conditions such as initial concentration of surfactant, ultrasonication period, and energy were kept identical for both cases). The final zeta potentials were measured for various trials and they were deduced as 68.8±9.3 mV and 51.8±9.5 mV in the absence and the presence of nonionic polymer respectively.

The charge balance equation is:

Total Zeta Pot. Pdot solution = Zeta Pot.(Pdot) + Zeta Pot. (empty CTAB);

Zeta Pot. of Empty CTAB: 68.8 ±9.3 mV

Zeta Pot. of Pdot: 51.8 ±9.5 mV

The calculated area under the Gaussian curves yielded the overlap percentage as 27.15 % and

the fraction of the nonionic polymer containing CTAB micelles was found to be 72%.


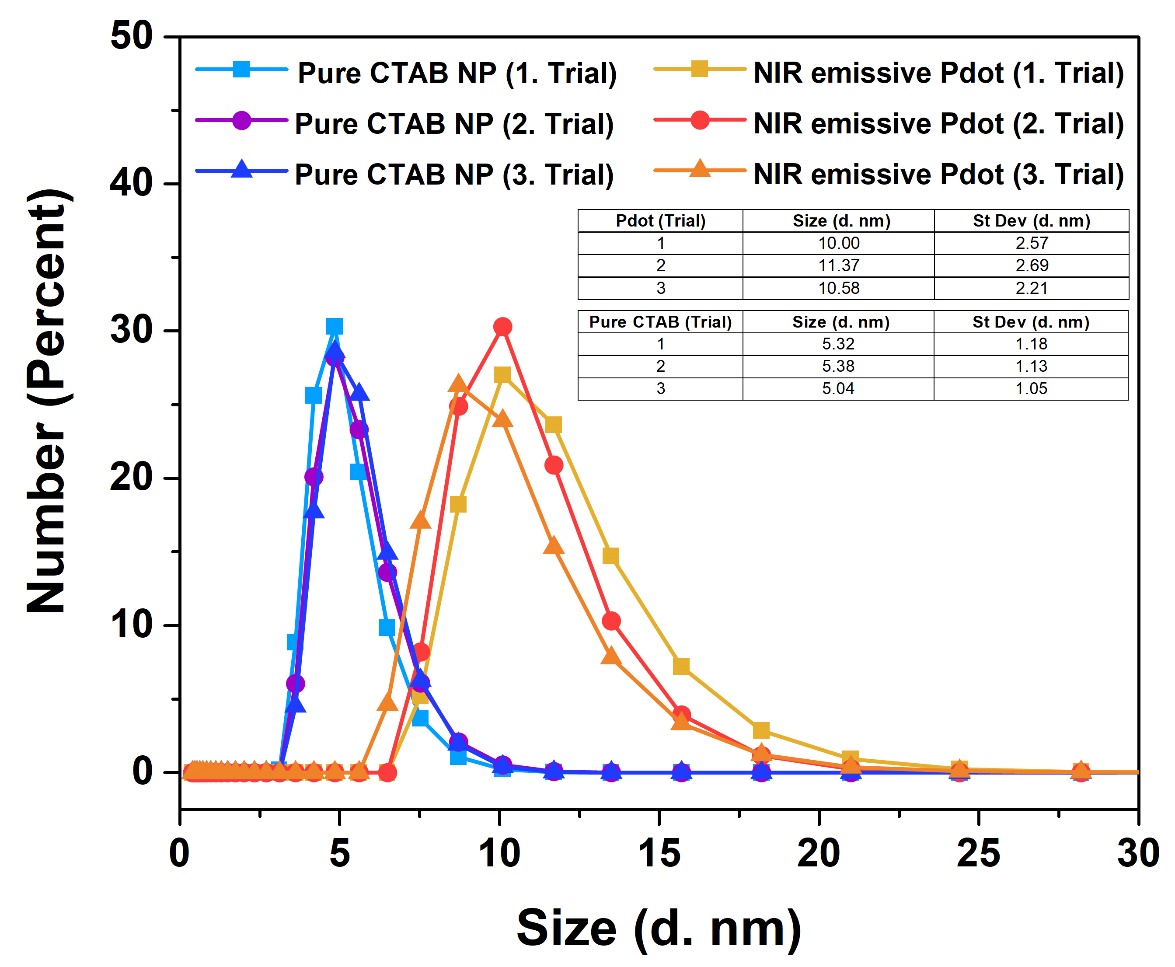


**Figure S12**. Analysis of pure CTAB nanoparticles (NPs) and the Pdots containing NIR emissive polymers in solution with the number-averaged size measurements.

Table S2. Size, zeta potential (ZP), mobility (Mob), and conductivity (Cond) values for the NIR emissive Pdots at different pH values and time points (5.5–7.5).


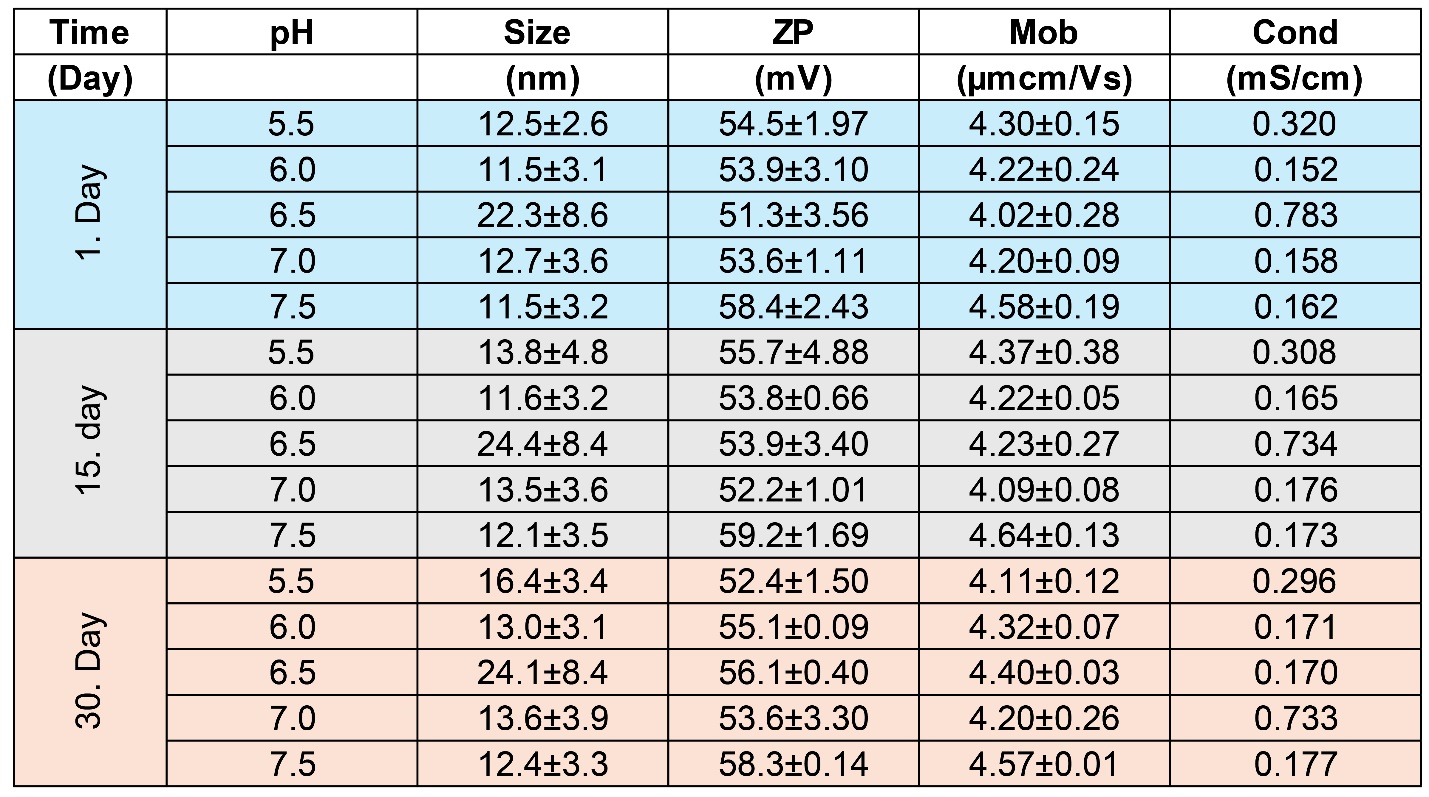


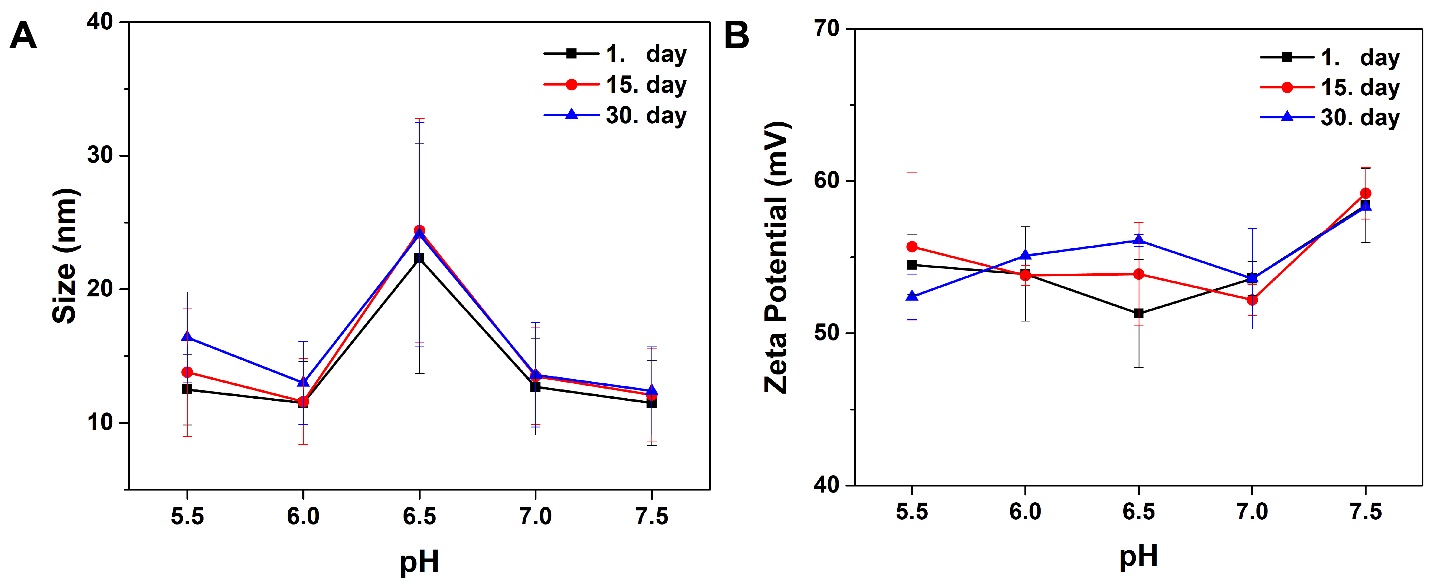


Figure S13. (A) Size values at different time points and pH range (5.5–7.5) graph for the NIR emissive Pdot; (B) Zeta potential values at different time points and pH range (5.5–7.5) graph for the NIR emissive Pdot.

**
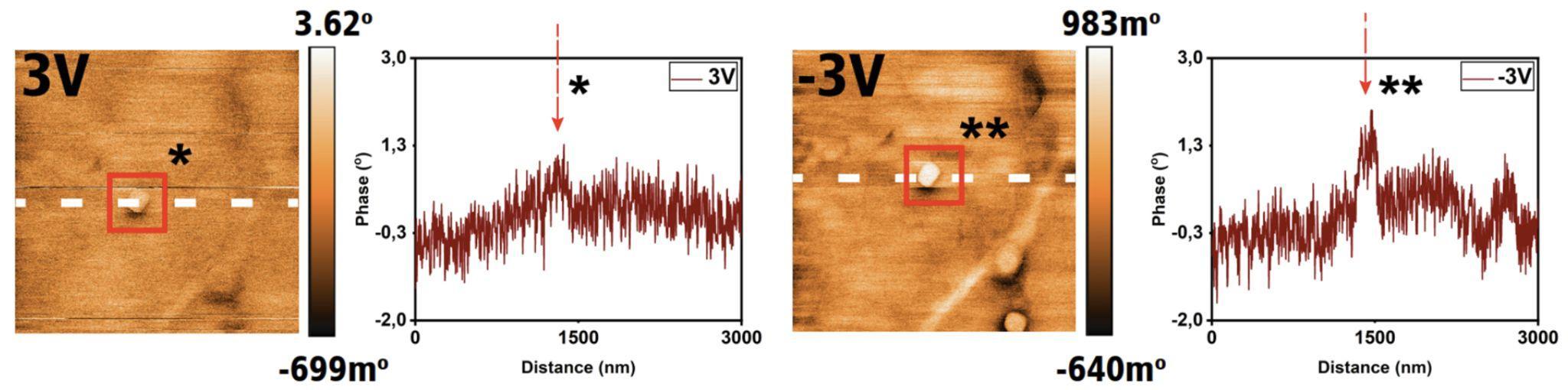
**

**Figure S14.** EFM Topography Images of NIR emissive Pdot at different voltage states and phase degree graphs respectively.


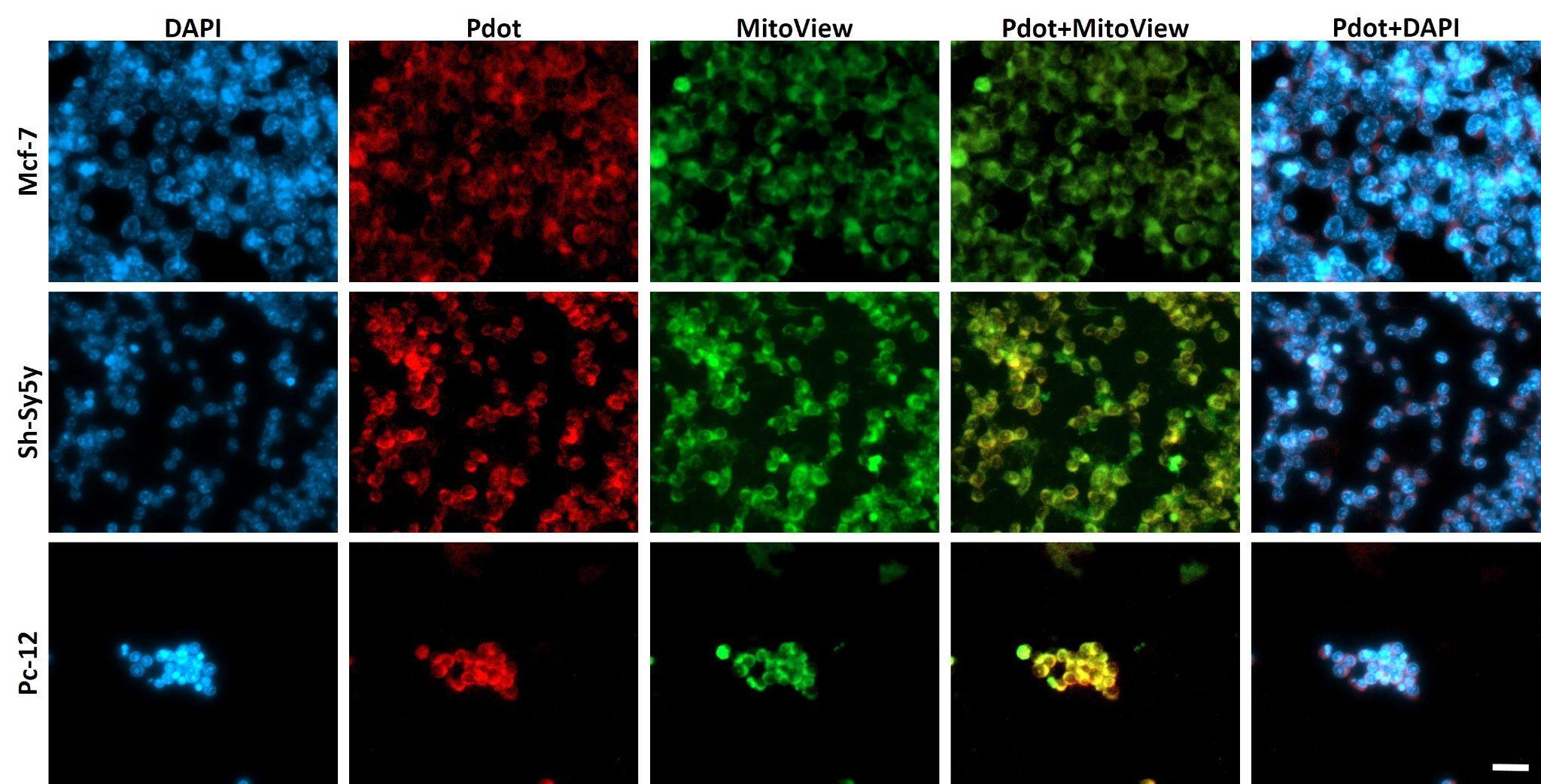


**Figure S15.** Fluorescence microscopy images of MCF−7, SH−SY5Y, and PC−12 cells, cultured with Pdot and labeled with DAPI and MitoView (blue: DAPI, red: Pdot, green: MitoView, scale bar: 20 µm).

**
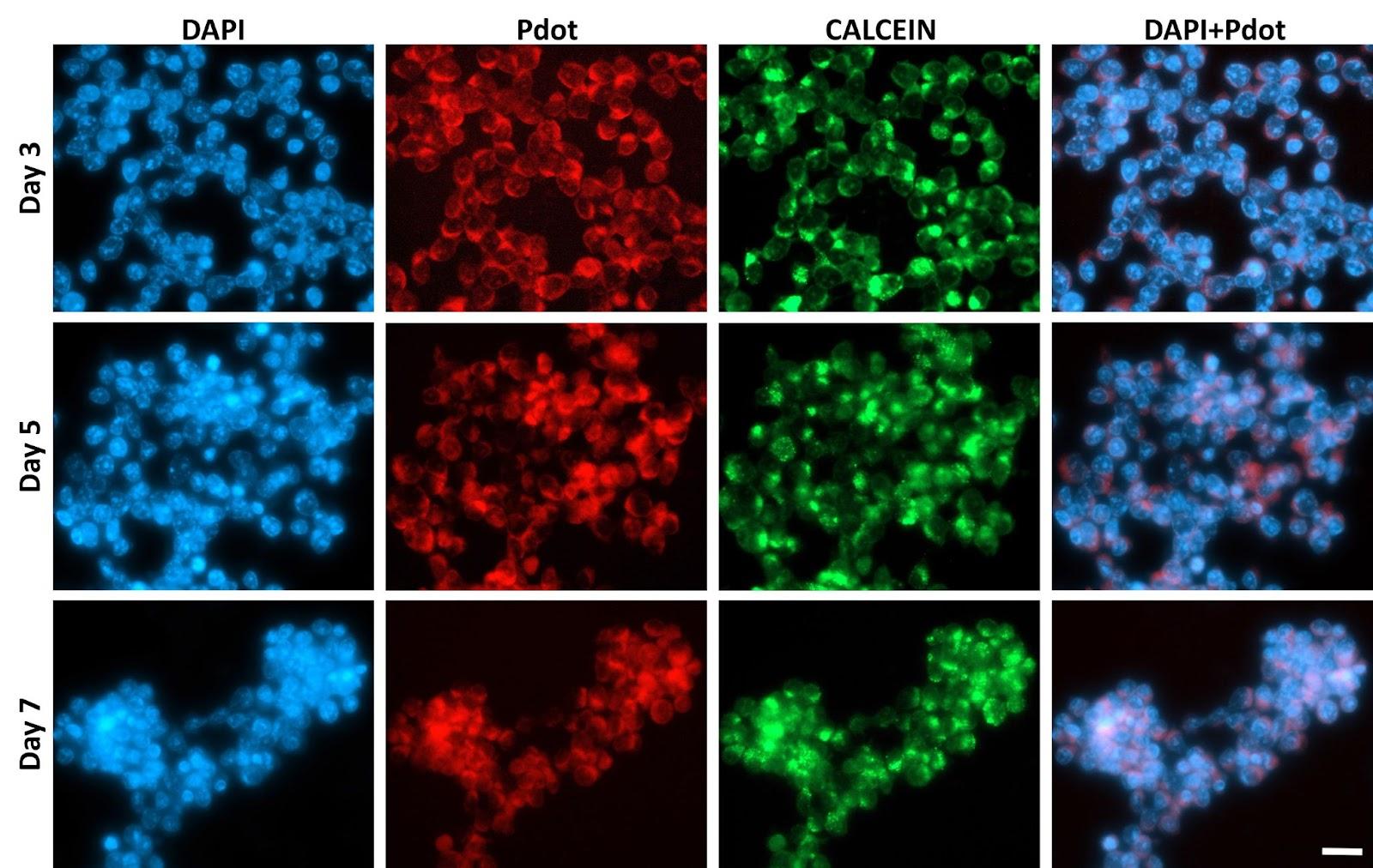
**

**Figure S16.** Fluorescence microscopy images of MCF−7 cells cultured with Pdot for 3, 5, and 7 days labeled with DAPI and Calcein Green (blue: DAPI, red: Pdot, green: Calcein, scale bar: 20 µm).

**
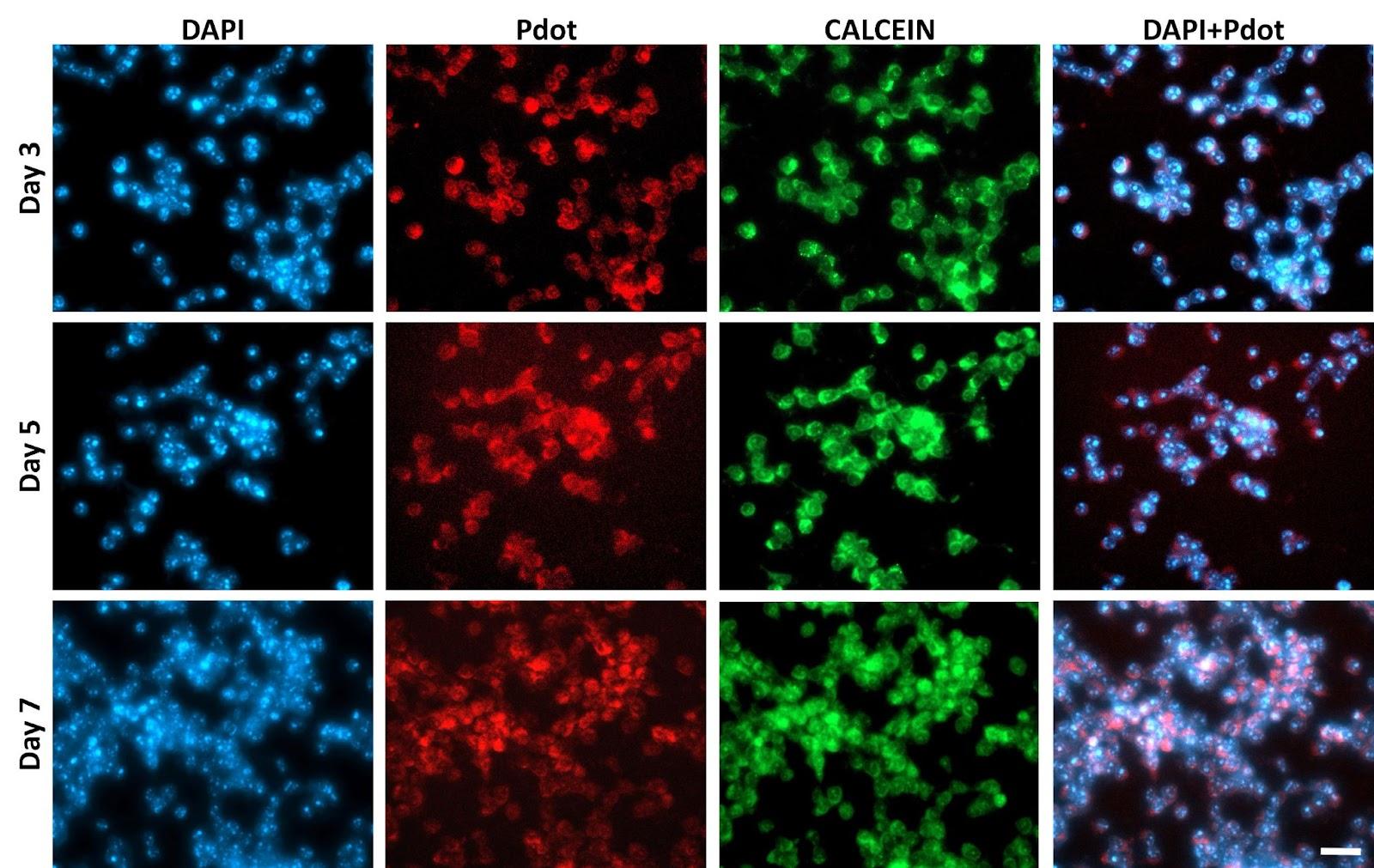
**

**Figure S17.** Fluorescence microscopy images of SH−SY5Y cells cultured with Pdot for 3, 5, and 7 days labeled with DAPI and Calcein Green (blue: DAPI, red: Pdot, green: Calcein, scale bar: 20 µm).

**
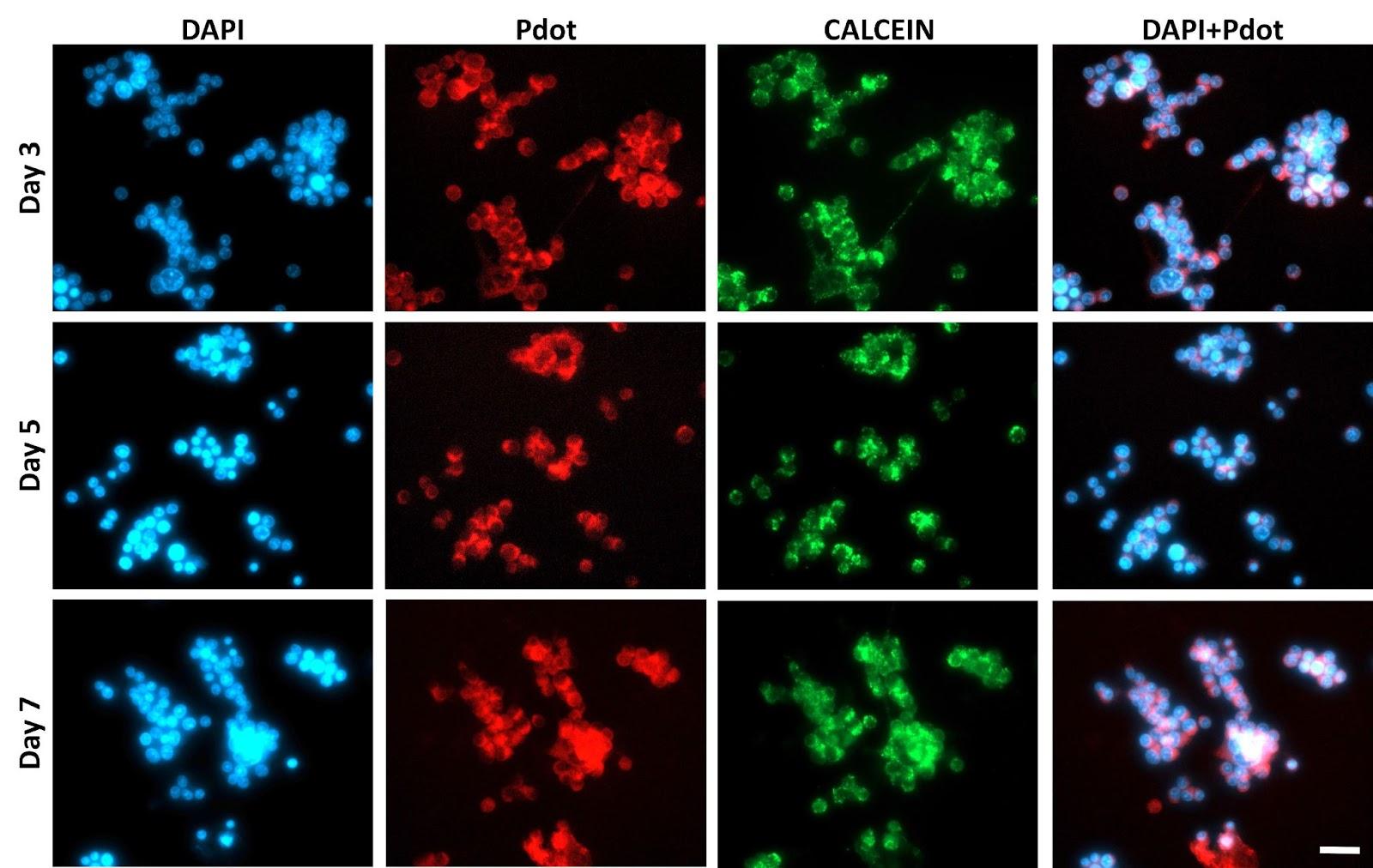
**

**Figure S18.** Fluorescence microscopy images of PC−12 cells cultured with Pdot for 3, 5, and 7 days labeled with DAPI and Calcein Green (blue: DAPI, red: Pdot, green: Calcein, scale bar: 20 µm).


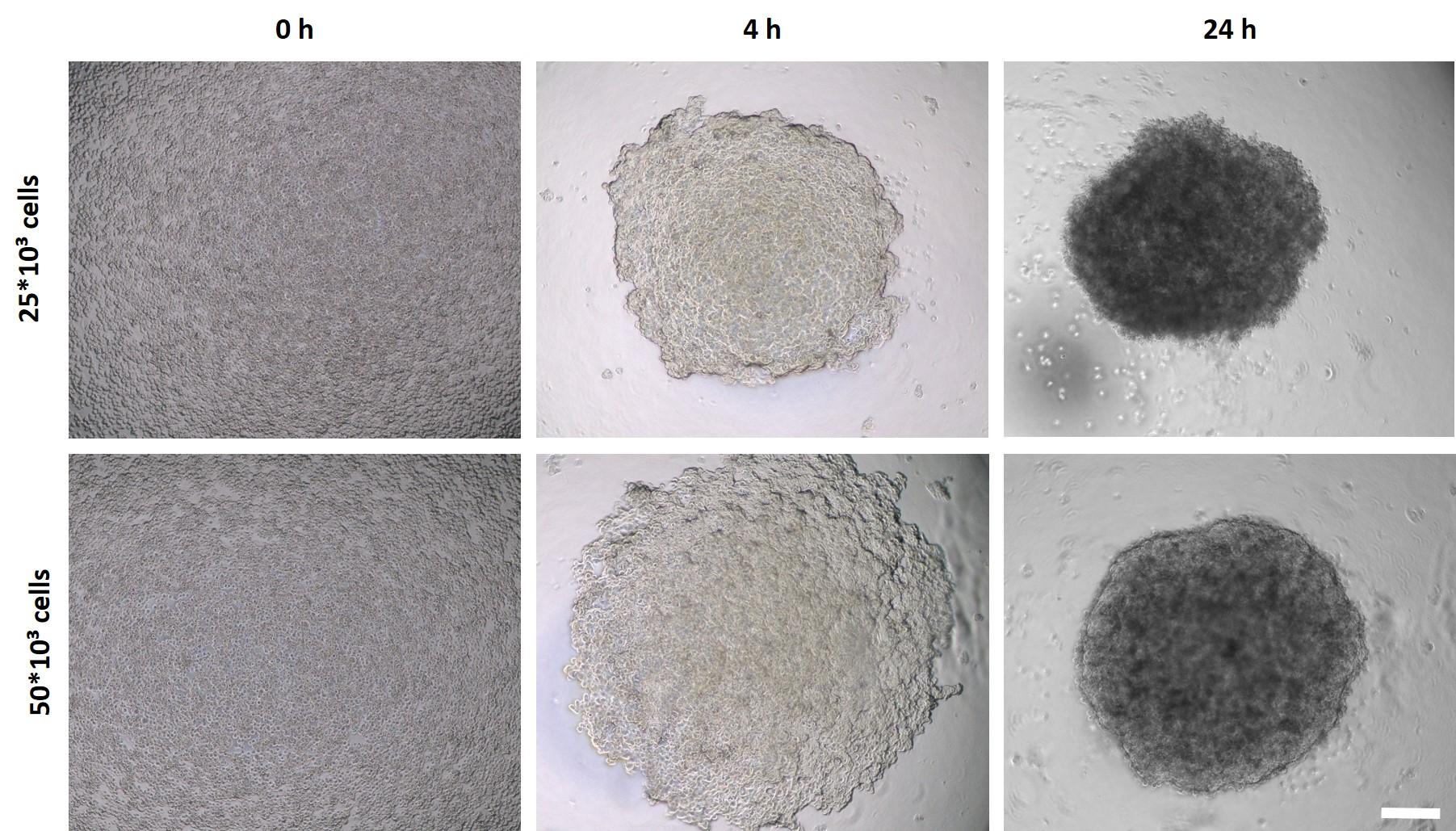


**Figure S19.** Light microscopy images of MCF−7 cell spheroids were obtained in 24 h via hanging drop methodology (scale bar: 200 µm).

**
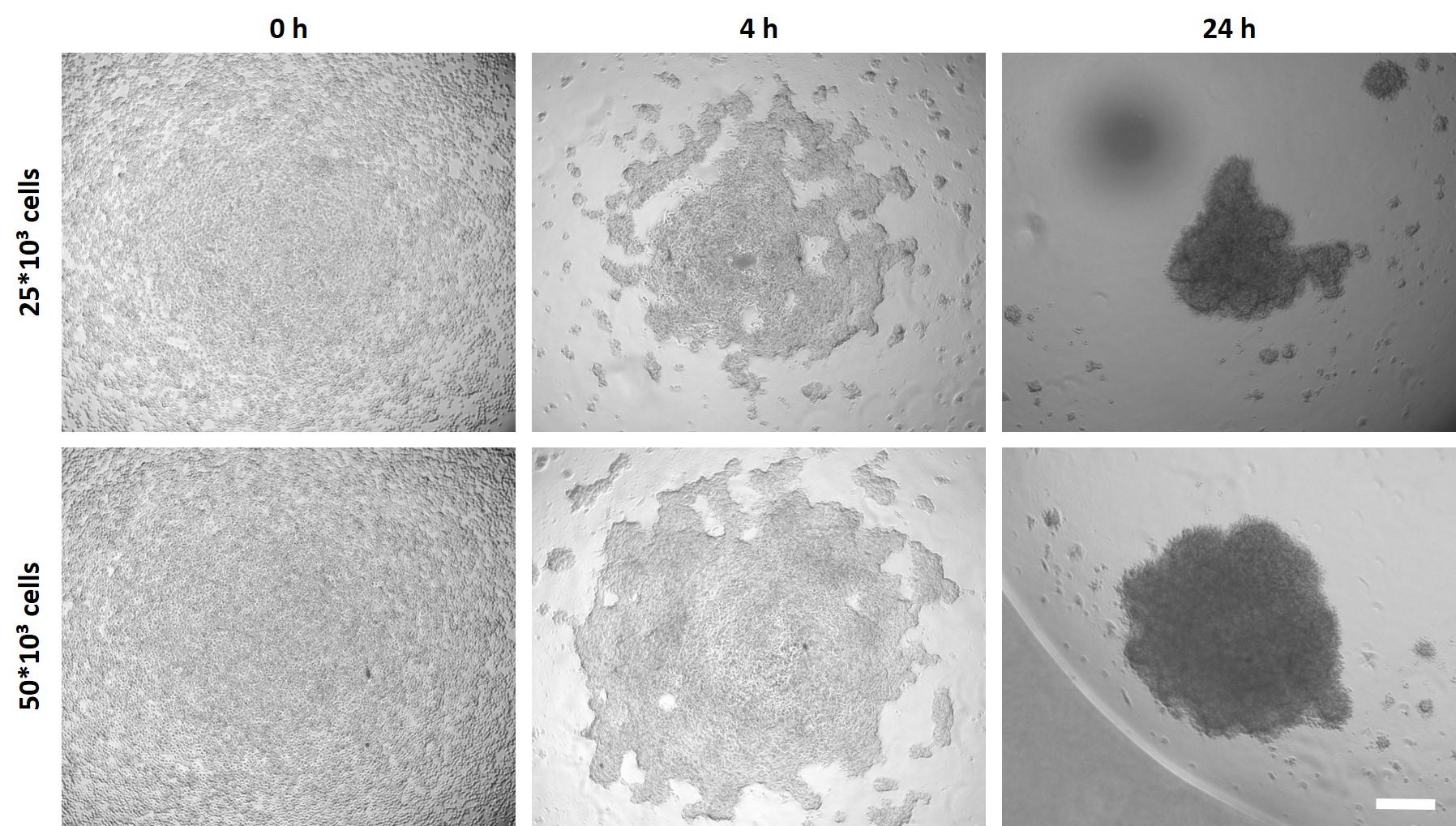
**

**Figure S20.** Light microscopy images of SH−SY5Y cell spheroids were obtained in 24 h via hanging drop methodology (scale bar: 200 µm)

**
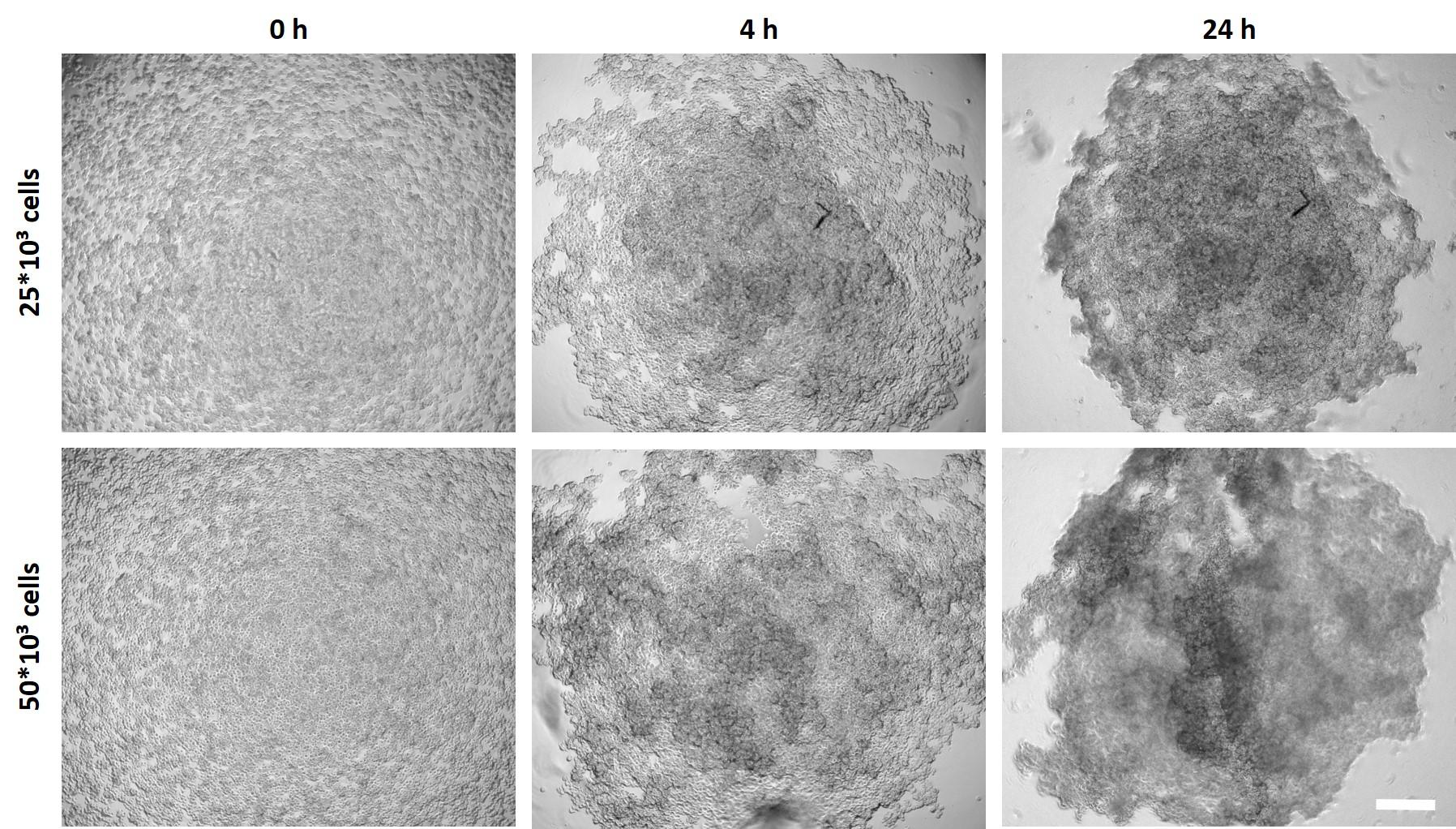
**

**Figure S21.** Light microscopy images of PC−12 cell spheroids were obtained in 24 h via hanging drop methodology (scale bar: 200 µm).


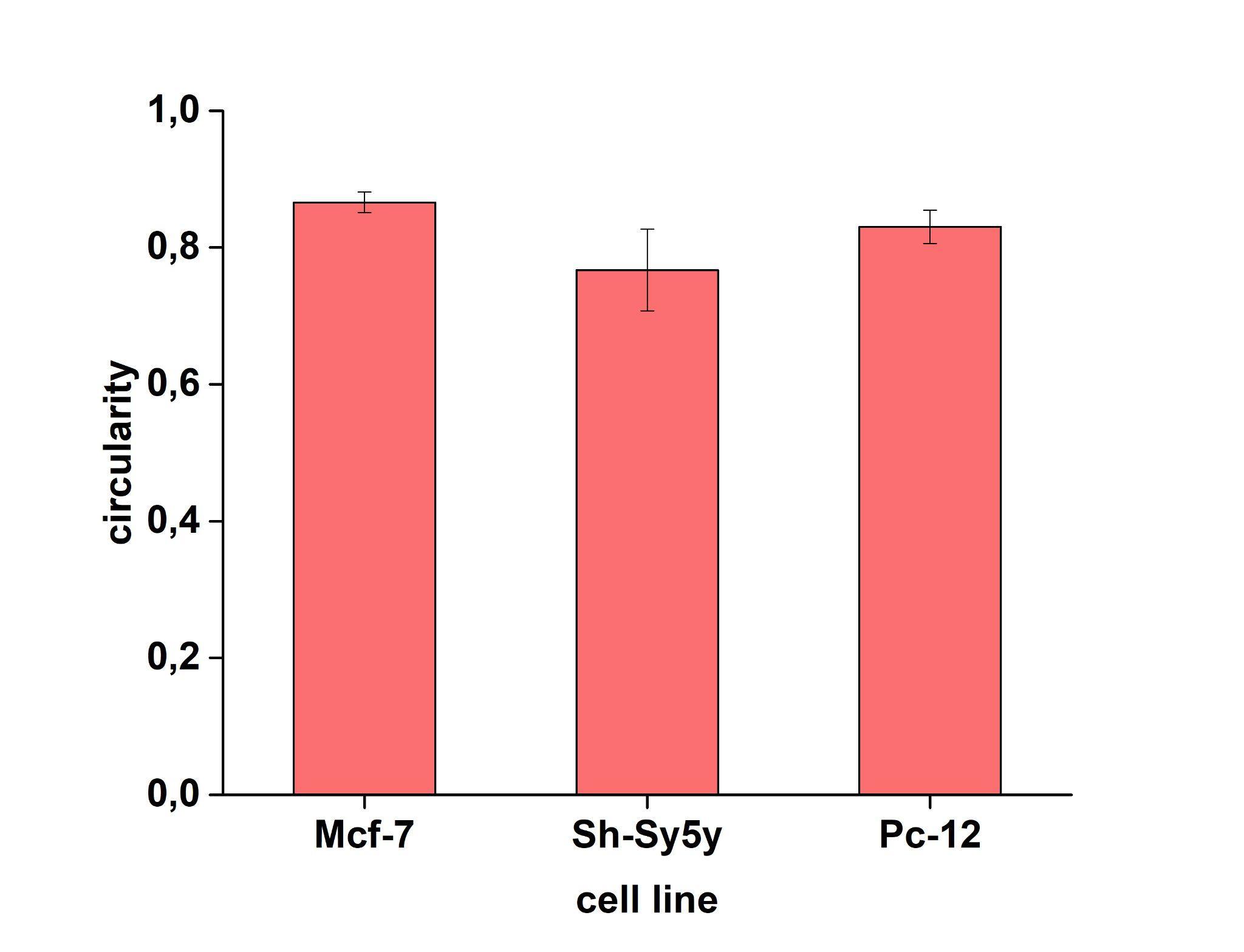


**Figure S22.** Circularity analysis of 3D cell spheroids obtained via hanging drop methodology.

**Karhunen−Loève Transform (KLT):**

KLT is a mathematical technique used for signal processing and feature extraction, particularly in the context of image compression and reconstruction. In the context of analyzing spheroids, KLT were used to extract specific features or characteristics of the Mitoview and Pdots distributions that are relevant for analysis. By KLT analysis we used fluorescence microscopy images in a more efficient manner while preserving important spatial information. The KLT analysis were performed by the FIJI software (IMAGE J2, version 2.14.0/1.54f ). Briefly: PNG file of microscopy images were exported to the FIJI software then images analyzed via Color Inspector 3D (v2.5). The axis C0, C1 and C2 and brightness, contrast, saturation and color rotation were kept by the default set values. As shown in figure S15, fixed area (14329 pixels) of spheroid images were analyzed via the FIJI Color Inspector 3D (v2.5). In summary, since our goal is to identify the main patterns or sources of variation in the distribution of Mitoview and Pdots within spheroids, KLT is worth exploring to show the colocalization in varying type of spheroid models.


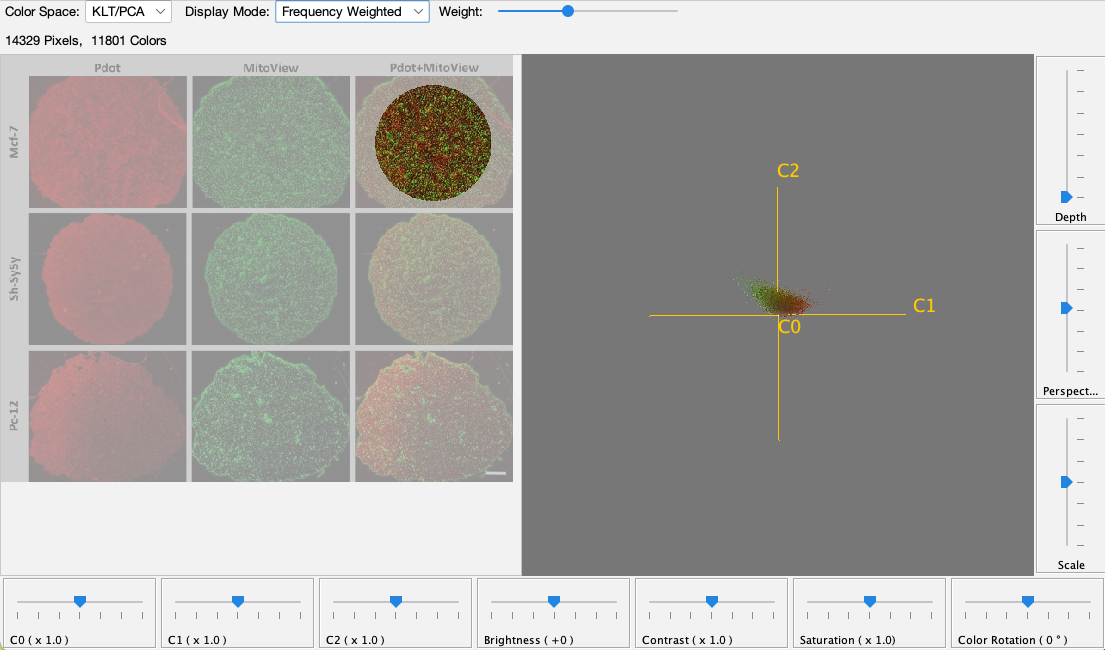

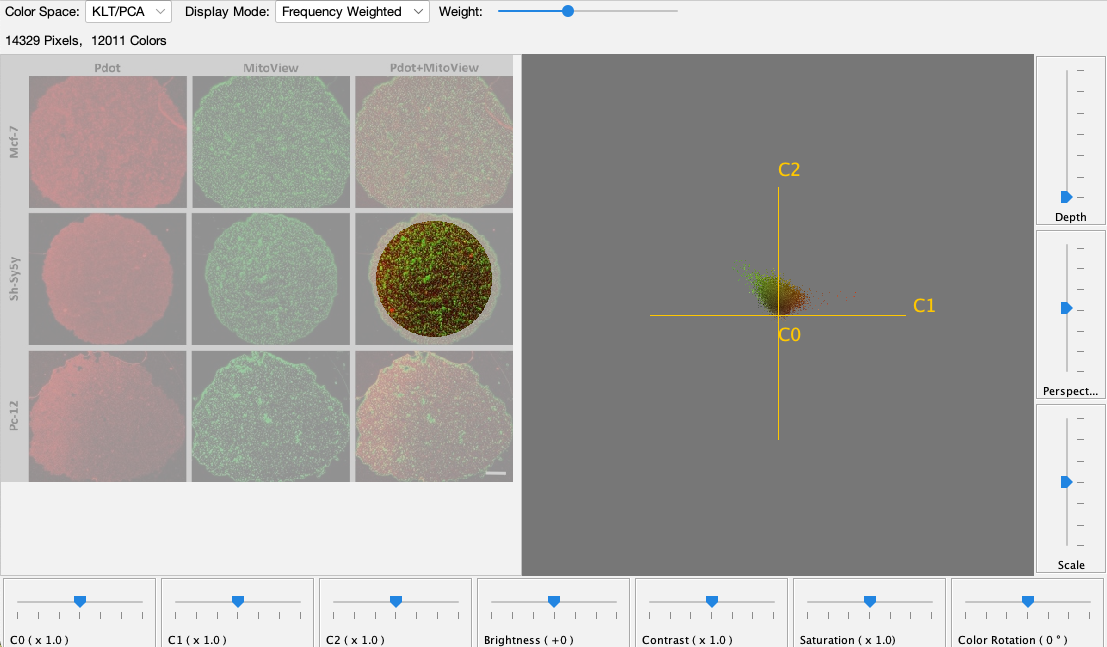

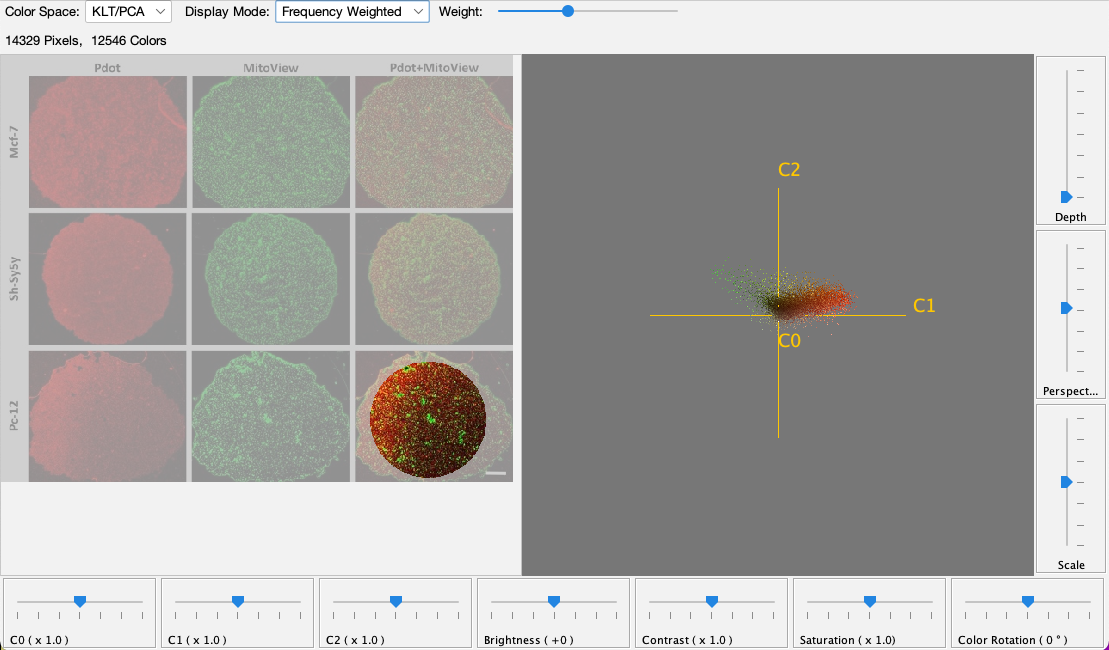


**Figure S23**. Screen shots of the FIJI Color Inspector 3D (v2.5) and Karhunen−Loève Transform (KLT) analysis cell spheroids.
